# Heatwave Exposure Accelerates Biological Aging via Metabolic Dysregulation

**DOI:** 10.34133/cbsystems.0602

**Published:** 2026-07-15

**Authors:** Dengyong Xu, Duanbin Li, Yao Chen, Bingjun Bai, Shangzhi Xie, Xing Gao, Guimei Wang, Shuting Cui, Wenbin Zhang, Jiapeng Bao, Henwei Huang, Binbin Xie

**Affiliations:** ^1^ Department of Colorectal Surgery, Sir Run Run Shaw Hospital, School of Medicine, Zhejiang University, Hangzhou 310016, PR China.; ^2^ Department of Cardiology, Sir Run Run Shaw Hospital, School of Medicine, Zhejiang University, Hangzhou 310016, PR China.; ^3^ Department of Medical Oncology, Sir Run Run Shaw Hospital, School of Medicine, Zhejiang University, Hangzhou 310016, PR China.; ^4^ Institute of Genomic Medicine, Wenzhou Medical University, Wenzhou 325035, PR China.; ^5^ Department of Oncology, The Second Affiliated Hospital of Soochow University, Jiangsu 215000, PR China.; ^6^ Department of Pulmonary and Critical Care Medicine, Regional Medical Center for National Institute of Respiratory Disease, Sir Run Run Shaw Hospital, Zhejiang University School of Medicine, Hangzhou 310016, PR China.; ^7^ Department of Orthopedic Surgery of the Second Affiliated Hospital, Zhejiang University School of Medicine, Hangzhou 310009, PR China.; ^8^ School of Electrical and Electronic Engineering and the LKC School of Medicine, Nanyang Technological University, Nanyang 639798, Singapore.; ^9^ Division of Gastroenterology, Hepatology and Endoscopy, Department of Medicine, Brigham & Women’s Hospital, Harvard Medical School, Boston, MA 02115, USA.

## Abstract

Heatwave (HW) exposure is increasing rapidly under climate change, yet its potential role in accelerating biological aging and the underlying mechanisms remain poorly understood. Leveraging data derived from the China Health and Retirement Longitudinal Study (CHARLS), a large population-based cohort in China, we examined whether exposure to HWs is linked to more rapid biological aging in adults of middle and advanced age. The Klemera–Doubal method (KDM) was applied to derive estimates of biological age (BA), and biological age acceleration (BAA) was calculated as biological age minus chronological age. HW exposure during the 12 months preceding BA assessments in 2011 and 2015 was quantified using 12 definitions based on different temperature threshold and duration. Longitudinal associations between HW exposure and BAA were evaluated using a difference-in-differences design. Among 2,318 participants (mean age, 58.7 years; 46.9% men), greater HW exposure was significantly associated with higher BAA. Under the most stringent (HW12; ≥4 consecutive days above the 97.5th percentile), each additional HW event and day increased BAA by 0.531 years [95% confidence interval (CI), 0.341 to 0.722] and 0.057 years (95% CI, 0.037 to 0.076). Stronger associations were observed among participants with body mass index ≥ 23 kg/m^2^, urban residents, and those living in southern or subtropical regions. HW exposure was also additionally associated with higher levels of total cholesterol and glycated hemoglobin A1c (HbA1c) levels. To explore potential biological mechanisms, transcriptomic profiling was performed in aged mice exposed to HW conditions. HW exposure induced 29 differentially expressed genes enriched in lipid metabolism and insulin resistance pathways, providing biological plausibility for the observed epidemiological associations. These results suggest that recurrent HW exposure may contribute to accelerated biological aging, potentially through metabolic disruption, and highlighting the vulnerability of aging populations to climate-related thermal stress and the need for targeted climate-adaptation strategies.

## Introduction

Population aging represents one of the most profound demographic shifts of the 21st century, with the number of people aged 60 years or older worldwide expected to exceed 2 billion by 2050 [1]. Aging involves a gradual decline of physiological resilience across multiple organ systems, which increases susceptibility to chronic diseases, functional impairment, and premature mortality [2]. Although chronological age serves as a conventional index of aging, it poorly reflects the marked interindividual heterogeneity in aging trajectories observed within populations. In contrast, biological age (BA), which integrates molecular, cellular, and clinical indicators of systemic physiological deterioration, has therefore emerged as a more informative and potentially reversible measure [3]. Among existing approaches, the Klemera–Doubal method (KDM) provides a robust and validated estimate of BA by combining clinical biomarkers capturing metabolic, inflammatory, cardiovascular, and renal function [4]. Discrepancies between biological and chronological age—referred to as biological age acceleration (BAA)—has been consistently linked to higher risks of cardiovascular disease, cognitive impairment, and mortality [5,6]. Importantly, while genetic factors account for only a modest proportion of interindividual variation in aging, environmental and contextual exposures appear to play a dominant role in shaping biological aging trajectories across the life course [1].

Among environmental stressors, heatwaves (HWs)—defined as prolonged periods of sustained, unusually high ambient temperatures—are becoming more frequent, are prolonged, and have intensified as a result of human-driven climate change [7]. The associated health burden is expected to escalate substantially; for example, in the United Kingdom alone, HW-related deaths may exceed 7,000 annually by 2050 [8]. A substantial body of epidemiological evidence has linked HW exposure to elevated risks of cardiovascular and respiratory mortality [9–13], cognitive dysfunction [14], and adverse mental health deterioration [15]. The impact is greater among older adults, driven by progressive losses in thermoregulatory function and physiological reserve with age [16]. Despite well-documented associations with acute morbidity and mortality, it remains largely unknown whether recurrent HW exposure contributes to the acceleration of systemic biological aging. Addressing this question is essential for understanding how climate-related thermal stress may shape long-term aging trajectories, and for informing adaptive public health strategies in rapidly aging societies.

This study investigated the potential role of HW exposure in accelerating biological aging among middle-aged and older adults in China at the national level. Leveraging repeated biomarker measurements, we quantified longitudinal changes in BA using the KDM algorithm and characterized HW exposure using multiple definitions that varied by temperature thresholds and event duration. To explore potential biological pathways, we conducted transcriptomic profiling of aging mice exposed to intermittent HW conditions. Advances in machine learning algorithms for multi-dimensional physiological signal processing—such as domain generalization and residual network architectures—have enabled robust feature extraction from complex biological data across diverse populations and contexts [17]. Building on these methodological developments, this study integrates population-level epidemiological evidence with molecular data to provide a multi-scale framework for understanding how climate-induced heat stress may accelerate biological aging through metabolic dysregulation.

## Methods

### Study population

This study utilized data based on the China Health and Retirement Longitudinal Study (CHARLS), a nationally representative, population-based cohort of Chinese adults aged 45 years or older, sampled from 150 counties and 450 villages distributed across China [18]. The baseline survey was conducted in 2011, followed by 4 subsequent waves in 2013, 2015, 2018, and 2020. Detailed descriptions of the study design, sampling procedures, and data collection methods have been reported previously [19].

Participants were eligible if they (a) were aged 45 years or older at the baseline and (b) had no missing values for the biomarker data used to BA estimation in both 2011 and 2015 assessments. Exclusion criteria included (a) missing meteorological data for HW exposure assessment; (b) severe renal insufficiency requiring hemodialysis; or (c) acute inflammation status [C-reactive protein (CRP) ≥10 mg/l], which could bias biomarker-based BA estimation.

**Fig. 1.**
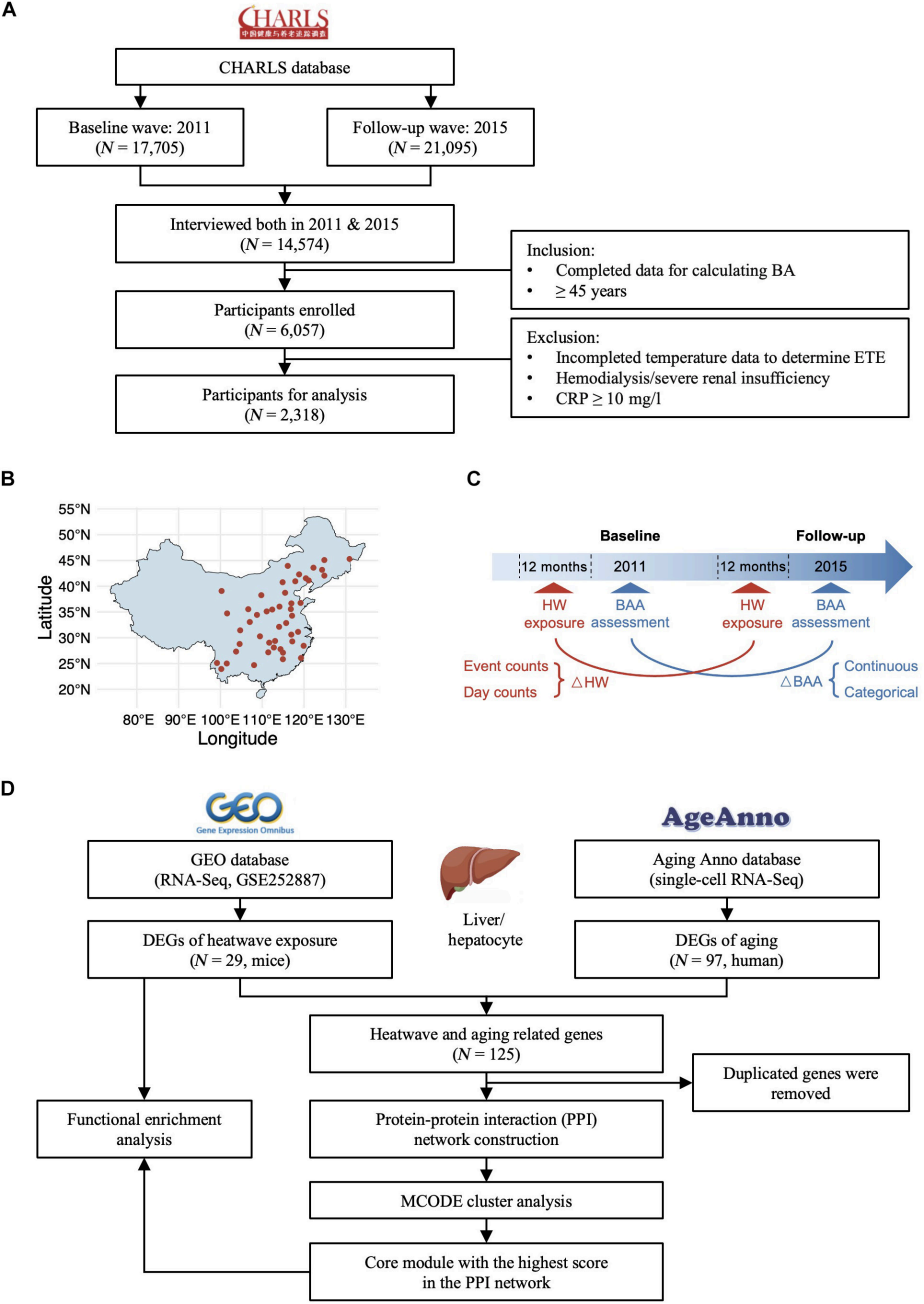
Study overview and analytical workflow. (A) Schematic flowchart depicting participant selection from the China Health and Retirement Longitudinal Study (CHARLS), including baseline (2011) and follow-up (2015) waves. (B) Geographic distribution of the CHARLS study population across 50 cities in China. (C) Illustration of the difference-in-differences (DiD) design evaluating the association between within-individual changes in HW exposure and biological age acceleration (BAA) from 2011 to 2015. (D) Transcriptomic analysis workflow integrating heatwave (HW)-related differentially expressed genes (DEGs) identified from liver tissue of aged mice and aging-related genes from the AgeAnno database.

A total of 2,318 participants met the inclusion criteria (Fig. 1A). Baseline characteristics were generally well balanced between included and excluded individuals (Table S1), as well as between participants from included versus excluded cities (Table S2), with most standardized mean differences below 0.1. The study protocol was approved by the Institutional Review Board of Peking University (IRB00001052-11015), and all participants provided written informed consent before participation.

### HW exposure

City-level HW exposure was assessed using daily meteorological data obtained from the National Center for Environmental Information (NCEI). Participants were assigned HW exposure based on the city of residence, assuming residential location remained relatively stable over the exposure assessment period. HW exposure was evaluated across 50 cities in China (Table S3), the geographic distribution of which is shown in Fig. 1B. Daily HW exposure was characterized by the heat index (°C), defined as the 24-h average apparent temperature.

In the absence of a universally accepted definition, HWs were operationalized using multiple combinations of temperature thresholds and event durations, consistent with prior epidemiological studies [9]. Thresholds were established based on city-level percentiles (90th, 92.5th, 95th, and 97.5th) of daily apparent temperature. HW events were defined as periods during which apparent temperature exceeded the specified threshold for at least 2, 3, or 4 consecutive days. For example, P95_4d denotes a HW defined as at least 4 consecutive days with daily apparent temperature at or above the 95th percentile. Complete definitions and the proportion of participants exposed under each HW definition are provided in Table S4. These multiple HW definitions, including the stringent HW12 metric (P97.5_4d), were specified a priori to evaluate robustness across exposure operationalizations rather than to select a single optimal definition.

For each participant, HW exposure was defined as the total number of HW days and HW events occurring during the 12 months preceding the baseline and follow-up assessments. In the difference-in-differences (DiD) analysis framework (Fig. 1C), ΔHW was derived by comparing HW exposure levels at baseline and follow-up.

### Biological age acceleration

BA was estimated using KDM, a validated regression-based algorithm that integrates a validated method that integrates multiple clinical biomarkers into a composite measure of physiological aging [20,21]. Briefly, KDM models the relationship between each biomarker and chronological age and combines the resulting parameters to generate a weighted estimate of BA while reducing the effects of measurement error and collinearity among biomarkers [4]. The formula is:

$$\text{Biological}\;\mathrm{age}=\frac{\sum_{i=1}^{n}\left(x_{i}-q_{i}\right)\frac{k_{i}}{s_{i}^{2}}+\frac{\mathrm{CA}}{S_{\mathrm{BA}}^{2}}}{\sum_{i=1}^{n}{\left(\frac{k_{i}}{s_{i}}\right)}^{2}+\frac{1}{s_{\mathrm{BA}}^{2}}}$$ (1)

where *x* denotes the biomarker value for an individual, and *s*, *q*, and *k* represent the root mean squared error, slope, and intercept from the corresponding regression model, respectively. CA stands for chronological age. The scaling factor sBA was defined as the square root of the variance in chronological age accounted for by the selected biomarkers. Biomarker selection followed previously validated implementations of the KDM, and biomarker weights were derived directly from regression parameters estimated within the model rather than assigned a priori.

Eight biomarkers were selected on the basis of prior validation in biological aging model [22,23], each representing a distinct physiological domain: inflammation (CRP, log-transformed), metabolic function [hemoglobin A1c (HbA1c), total cholesterol (TC), triglycerides (TG)], renal function [blood urea nitrogen (BUN), serum creatinine (SCR)], hematological status [platelet count (PLT)], and cardiovascular health [systolic blood pressure (SBP)]. These biomarkers collectively represent multiple physiological domains, including inflammatory, metabolic, cardiovascular, renal, and hematologic systems, supporting a multi-system assessment of biological aging.

BAA was calculated as BA minus chronological age at baseline (2011) and follow-up (2015). The change in BAA (ΔBAA) was defined as the difference between follow-up and baseline BAA such that positive values correspond to accelerated aging and negative values correspond to decelerated aging. ΔBAA was analyzed both continuously and categorically (ΔBAA ≥ 0 versus < 0), with the continuous measure as the primary outcome and the dichotomized measure used in secondary analyses for ease of interpretation. Changes in individual BA-related biomarkers were also examined.

### Covariates

Baseline covariates were obtained from CHARLS Wave 2011. Demographic variables included age (continuous, years), gender (male or female), and body mass index (BMI; continuous, kg/m^2^). Marital status was categorized as married or other (including never married, widowed, divorced, separated, or cohabiting). Educational attainment was classified as illiterate, literate but below elementary school, or elementary school and above. Income status was defined based on whether participants reported receiving wage or bonus income in the past year (yes or no).

Location-related covariates included geographical region (northern or southern China, defined by the Qinling–Huaihe line), climate zone (subtropical monsoon, temperate monsoon, temperate continental, or plateau mountain), and residence type (urban or rural). Residential status was determined according to the official household registration (hukou) classification recorded in the CHARLS database, which reflects long-term residential location and is consistent with national census definitions.

Lifestyle factors included the status of smoking (never, former, or current) and alcohol consumption (never, less than once monthly, or more than once monthly). Health-related covariates included self-reported diabetes and hypertension (each classified as yes or no), as well as depressive symptoms assessed using the Center for Epidemiologic Studies Depression 10-item scale (CESD-10) [24]. CESD-10 scores range from 0 to 30, with increasing values corresponding to more severe depressive symptoms.

Time-varying covariates from 2011 to 2015 included changes in drinking and smoking status (categorized as no to yes, yes to no, or unchanged), change in CESD-10 score (continuous), and weight change (≥5 kg gain or loss, or unchanged). Covariate selection was informed by a directed acyclic graph (Fig. S1) to minimize confounding between ΔHW and ΔBAA.

### Transcriptomic analysis in aging mice

To complement the epidemiologic findings linking HW exposure to accelerated biological aging, we explored potential molecular mechanisms using transcriptomic data from HW-exposed aging mice (workflow summarized in Fig. 1D). Liver transcriptomic profiles from aged C57BL/6J mice subjected to periodic HW exposure were derived from the Gene Expression Omnibus (GEO) database; details are provided elsewhere [25]. The edgeR package was used to perform differential gene expression analysis, with normalization performed using the trimmed mean of *M*-values (TMM) method and modeling via generalized linear models. Significantly differentially expressed genes (DEGs) were identified using thresholds of false discovery rate (FDR) < 0.05 and |log_2_FC| > 1. Heatmaps were used to visualize expression patterns.

To evaluate aging relevance, HW-responsive genes were intersected with aging-related genes curated from the AgeAnno database, which identifies age-associated genes based on single-cell RNA sequencing of human hepatocytes comparing old versus middle-aged individuals. Overlapping genes were identified as potential mediators of HW-induced biological aging.

Protein–protein interactions (PPIs) among the combined gene list were retrieved from STRING (v11.5; *Homo sapiens*), applying a medium confidence threshold (interaction score > 0.400), followed by identification of highly connected modules using the MCODE plugin in Cytoscape (v3.9.1). The clusterProfiler R package was used to perform functional enrichment analysis, covering Gene Ontology (GO) categories and Kyoto Encyclopedia of Genes and Genomes (KEGG) pathways.

### Statistical analyses

Descriptive analyses were performed to characterize the study population, with continuous variables summarized as mean ± SD or median [interquartile range (IQR)] and categorical variables as frequencies and proportions. Missing values were imputed using the multiple imputation by chained equations (MICE) approach. To evaluate the longitudinal consistency of the BA construct, Pearson correlations were calculated between Ln-transformed biomarkers at both time points.

Cross-sectional associations between the number of HW exposure days and BAA were examined using generalized linear models. Longitudinal associations between changes in HW exposure and changes in BAA were analyzed using a DiD framework, which compares within-personal changes between baseline and follow-up, thereby enhancing causal inference [26,27]. The same DiD approach was used to examine the relationship between changes in HW exposure and alterations in BA components. Generalized linear models were fitted within the DiD framework, adjusting for baseline covariates and time-varying covariates as specified. The Benjamini–Hochberg method was used to control the FDR, and results are reported with both nominal and adjusted *P* values.

Subgroup analysis was performed by age stratification (<50, 50 to 59, or ≥60 years), gender (female or male), residence (rural or urban), BMI (<23 or ≥23 kg/m^2^), geographic region (northern or southern China), and climate zones (subtropical monsoon zone, temperate monsoon zone, temperate continental zone, or plateau mountain climate zone). Effect modification was assessed by including interaction terms in the models, with *P* values for interaction obtained based on likelihood ratio tests contrasting models that included interaction terms with those that did not.

A series of sensitivity analyses was undertaken to evaluate the stability of the study findings. These included (a) mixed-effects models including random intercepts for 4 region–residence strata defined by region (north versus south) and urbanicity (rural versus urban) to account for underlying heterogeneity at area level; (b) exclusion of outliers with extreme ΔBAA (>5 or >10 years); and (c) exclusion of participants with minimal change in BAA (<1 year) to reduce noise.

All analyses were carried out using R version 4.4.1, and statistical significance was assessed using a 2-tailed threshold of *P* < 0.05.

### Data availability

The individual-level demographic, biomarker, and health data were obtained from the CHARLS, which is publicly available upon application at http://charls.pku.edu.cn/. City-level meteorological data for HW exposure assessment were retrieved from the NCEI of the National Oceanic and Atmospheric Administration (NOAA) (https://www.ncei.noaa.gov/access). Transcriptomic data from HW-exposed aging mice were obtained from the GEO under accession number GSE252887.

## Results

### Population characteristics

The study population comprised 2,318 adults aged ≥45 years (mean age, (58.7 ± 8.7) years), of whom 53.1% were female. Most participants resided in rural areas (69.2%) and in southern China (57.5%) (Table 1). Educational attainment was generally low, with 27.4% being illiterate. At baseline, 30.2% were current smokers and 8.1% reported alcohol consumption more than once per month. The prevalence of self-reported hypertension and diabetes was 24.4% and 4.7%, respectively. During follow-up, 9.3% experienced weight gain of ≥5 kg. Changes in lifestyle behaviors were also observed: 8.0% initiated alcohol consumption, 8.4% discontinued alcohol consumption, 1.7% initiated smoking, and 6.0% quit smoking (Table 1).

**Table 1.** Baseline characteristics and changes from 2011 to 2015. Categorical variables are presented as *n* (%), and continuous variables are presented as mean ± SD or median [IQR]. The CESD-10 scale was used to measure depressive symptoms.

Characteristics	Overall (*n* = 2,318)
Baseline characteristics (2011)	
Age, years	58.67 ± 8.65
Gender, *n*/%	
Female	1,231 (53.1)
Male	1,087 (46.9)
Body mass index/(kg·m^-2^)	23.41±3.78
Education attainment, *n*/%	
Illiterate	634 (27.4)
Literate	449 (19.4)
Elementary school and above	1,235 (53.3)
Income status, *n*/%	
No	2,049 (88.4)
Yes	269 (11.6)
Residence, *n*/%	
Urban	715 (30.8)
Rural	1,603 (69.2)
Region, *n*/%	
Southern China	1,334 (57.5)
Northern China	984 (42.5)
Climate zones, *n*/%	
Subtropical monsoon zone	465 (20.1)
Temperate monsoon zone	796 (34.3)
Temperate continental zone	185 (8.0)
Plateau mountain climate zone	872 (37.6)
Marital status, *n*/%	
Married	2,068 (89.2)
Others	250 (10.8)
Smoking status, *n*/%	
Never	1,410 (60.8)
Quit	207 (8.9)
Current	701 (30.2)
Drinking status, *n*/%	
Never	1,494 (64.5)
Less than once a month	637 (27.5)
More than once a month	187 (8.1)
Diabetes, *n*/%	
No	2,208 (95.3)
Yes	110 (4.7)
Hypertension, *n*/%	
No	1,753 (75.6)
Yes	565 (24.4)
Depression score	8.0 [4.0, 13.0]
Characteristics change (from 2011 to 2015)	
Depressive symptoms change	0.0 [−4.0, 3.0]
Drinking, *n*/%	
No change	1,938 (83.6)
From yes to no	195 (8.4)
From no to yes	185 (8.0)
Smoking, *n*/%	
No change	2,139 (92.3)
From yes to no	139 (6.0)
From no to yes	40 (1.7)
Weight change (≥5 kg), *n*/%	
No change	1,945 (83.9)
Weight gain	215 (9.3)
Weight loss	158 (6.8)
ΔBAA change (from 2011 to 2015)	
Continuous	0.93 [−1.67, 3.59]
Categorical ΔBAA	
ΔBAA ≥ 0 (accelerated)	1,363 (58.8)
ΔBAA < 0 (delayed)	955 (41.2)

SD, standard deviation; IQR, interquartile range; CESD-10, 10-item Center for Epidemiologic Studies Depression Scale; ΔBAA, change in biological age acceleration

### Trends in HW exposure and biological aging

Between 2011 and 2015, HW exposure showed a slight overall decline. Under the HW12 definition (P97.5_4d), the mean number of HW events decreased by 0.36, and the cumulative duration shortened by 2.68 d (Table S5).

During the same period, the mean BA increased from 57.3 to 62.3 years (Fig. S2A). The median BAA rose slightly from −0.72 to 0.25 years, with an average ΔBAA of 0.93 years (IQR: −1.67, 3.59) between the 2 time points (Table S6 and Fig. S2B), indicating a population-level trend of increased physiological aging at the population level.

Overall, 58.8% of participants exhibited accelerated biological aging (ΔBAA ≥ 0), whereas 41.2% showed delayed aging (ΔBAA < 0). Baseline characteristics were largely comparable between these 2 groups (Table S7). Additionally, the core biomarkers contributing to the BA algorithm remained positively correlated between the 2011 and 2015 assessments, indicating the longitudinal consistency of the BA construct (Fig. S3). Despite a modest decline in mean HW exposure at the population level, individual-level changes varied substantially across regions, providing sufficient within-person variation for the DiD analysis.

### Association between baseline HW days and BAA

In the cross-sectional analyses, baseline HW exposure was positively associated with BAA; however, both the magnitude and statistical significance of the associations varied according to the HW definition used (Fig. S4). For example, under the HW12 (P97.5_4d), each additional day of HW exposure was associated with a 0.160-year increase in BAA [95% confidence interval (CI), 0.021 to 0.300; FDR-adjusted *P* = 0.040]. In contrast, several alternative HW definitions, including HW03, HW09, and HW10, showed no statistically significant associations with BAA (Table S8).

### Association between ΔHW and ΔBAA

**Fig. 2.**
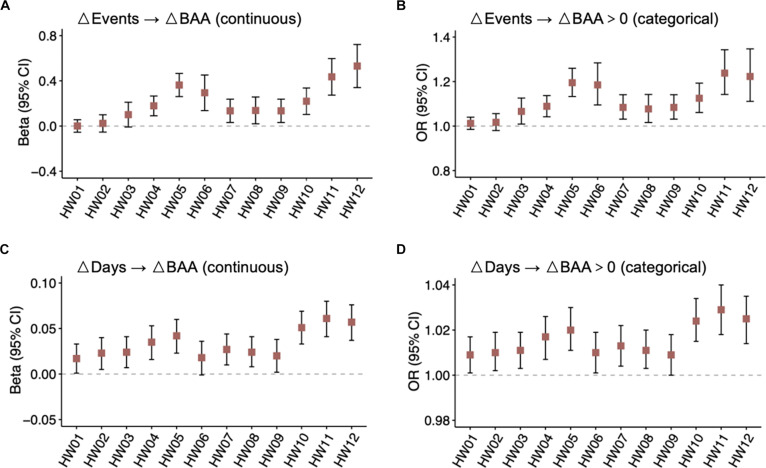
DiD estimates for the associations between HW changes and BAA (*n* = 2,318). Associations were assessed between within-individual changes in HW (ΔHW) exposure from 2011 to 2015 and changes in BAA (ΔBAA), using a DiD analytical framework. HW exposure was defined by event frequency (A and B) or total days (C and D). Outcomes included continuous ΔBAA (A and C) and binary ΔBAA > 0 (B and D). Effect estimates are expressed as beta coefficients (years per HW event/day) and odds ratios (ORs), along with 95% CIs. Models included adjustments for baseline covariates—age, sex, BMI, marital status, education, residence, region, economic status, smoking, drinking, depressive symptoms, hypertension, and diabetes—as well as time-varying factors reflecting changes in depression, drinking, smoking, and weight.

Within the DiD framework, we examined the association between ΔHW (both event counts and day counts) and ΔBAA (modeled as both a continuous and a categorical outcomes) (Fig. 2). In model 3, the fully adjusted model, each additional HW12 event was associated with a higher BAA, with an estimated increase of 0.531 years (95% CI, 0.341 to 0.722; FDR-adjusted *P* < 0.001); each additional HW12 day was associated with a further 0.057-year increase (95% CI, 0.037 to 0.076; FDR-adjusted *P* < 0.001) (Table S9). When ΔBAA was modeled categorically (ΔBAA ≥ 0 versus < 0), similar patterns were observed: each additional HW12 event was associated with 22.3% higher odds of accelerated biological aging [odds ratio (OR): 1.223, 95% CI: 1.111 to 1.347, FDR-adjusted *P* < 0.001]; additionally, each HW12 day was associated with increased odds of 2.5% (OR, 1.025; 95% CI, 1.014 to 1.035; FDR-adjusted *P* < 0.001) (Table S10).

Across HW definitions, effect estimates tended to increase with more stringent definitions, suggesting stronger associations for more intense and prolonged HW exposure. The direction of the associations was consistent across the unadjusted (model 1), baseline-adjusted (model 2), and fully adjusted models (Tables S9 and S10).

### Sensitivity analyses

The main findings were robust across multiple sensitivity analyses. First, a mixed-effects model incorporating random intercepts at the area level yielded consistent findings (Table S11). Second, the results remained consistent after excluding participants with extreme changes in BAA (Tables S12 and S13). Finally, excluding participants with minimal changes in BAA (|ΔBAA| < 1) also did not alter the main findings (Table S14).

### Subgroup analyses

Heterogeneous associations between ΔHW and ΔBAA were observed in the subgroup analyses (Table 2). Stronger associations were observed among participants with BMI ≥ 23 kg/m^2^ (*P* for interaction = 0.017 for HW days; 0.003 for HW events), urban residents (both *P* for interaction < 0.001), those living in southern China (*P* for interaction = 0.019 for HW days; 0.017 for HW events), and those residing in subtropical monsoon zone (both *P* for interaction < 0.001). No significant effect modification was observed by age group or sex.

**Table 2.** Subgroup analyses of the association between changes in HW exposure and BAA. The table summarizes adjusted beta estimates with 95% CIs for subgroup-specific associations between changes in HW exposure and BAA. Generalized linear models accounted for covariates, consistent with the main analysis. Interaction *P* values were derived using the likelihood ratio test to assess overall subgroup differences.

Subgroup	Subject	HW days			HW events		
		Beta	95% CI	*P* for interaction	Beta	95% CI	*P* for interaction
Age/years				0.718			0.484
<50	439	0.074	(0.025, 0.122)		0.643	(0.185, 1.100)	
50–59	864	0.071	(0.037, 0.106)		0.723	(0.403, 1.044)	
≥60	1,015	0.049	(0.021, 0.077)		0.440	(0.157, 0.723)	
Gender				0.355			0.158
Female	1,231	0.065	(0.039, 0.091)		0.657	(0.398, 0.916)	
Male	1,087	0.048	(0.018, 0.078)		0.405	(0.119, 0.692)	
Body mass index, /(kg·m^-2^)				0.017			0.003
<23	1,178	0.034	(0.009, 0.060)		0.255	(−0.001, 0.510)	
≥23	1,140	0.082	(0.052, 0.112)		0.836	(0.548, 1.125)	
Residence				<0.001			<0.001
Rural	1,603	0.023	(−0.002, 0.049)		0.152	(−0.084, 0.389)	
Urban	715	0.108	(0.077, 0.139)		1.300	(0.967, 1.632)	
Geographic region				0.019			0.017
Northern China	984	0.030	(−0.047, 0.108)		0.271	(−0.249, 0.79)	
Southern China	1,334	0.065	(0.044, 0.086)		0.623	(0.407, 0.839)	
Climate zones				<0.001			<0.001
Subtropical monsoon zone	465	0.136	(0.096, 0.176)		1.298	(0.916, 1.680)	
Temperate monsoon zone	796	−0.078	(−0.217, 0.062)		0.583	(−0.280, 1.446)	
Temperate continental zone	185	−0.640	(−1.367, 0.086)		−2.561	(−5.467, 0.344)	
Plateau mountain climate zone	872	0.024	(−0.005, 0.052)		0.124	(−0.158, 0.407)	

### Association between ΔHW and ΔBAA component biomarkers

**Fig. 3.**
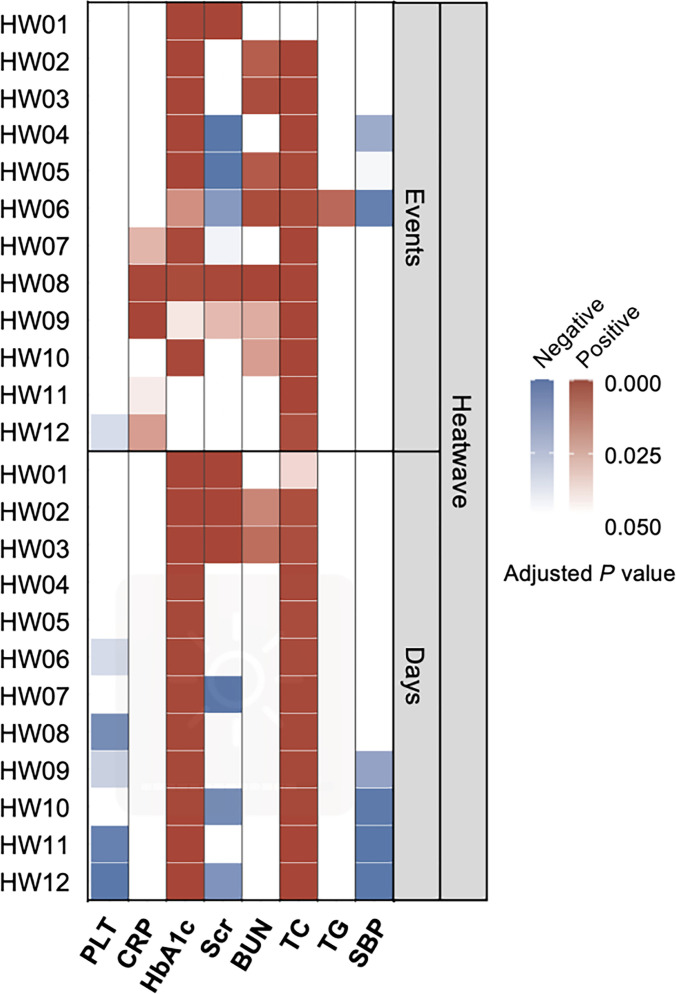
Associations between HW changes and BA-related biomarkers (*n* = 2,318). Heatmap showing associations between changes in HW exposure—quantified by the number of events and total days—and changes in individual biomarkers used to calculate BAA. Associations were estimated using generalized linear models within a DiD framework after accounting for baseline characteristics and covariates that changed over time. Associations are displayed only if the FDR-adjusted *P* value is <0.05. Red, positive associations; blue, negative associations; darker shading represents stronger statistical significance. Biomarkers include C-reactive protein (CRP), platelet count (PLT), glycated hemoglobin (HbA1c), serum creatinine (SCR), blood urea nitrogen (BUN), total cholesterol (TC), triglycerides (TG), and systolic blood pressure (SBP).

Fig. 3 presents a heatmap summarizing the statistically significant associations between changes in ΔHW and changes in the individual biomarkers contributing to BAA, with detailed estimates provided in Table S15. Across definitions, HW exposure was consistently associated with increases in TC and HbA1c. Under the HW12 definition (P97.5_4d), each additional day of HW exposure was associated with a 0.154 mg/dl increase in TC (95% CI: 0.007 to 0.300, FDR-adjusted *P* = 0.039) and a 0.008% rise in HbA1c (95% CI: 0.005 to 0.011, FDR-adjusted *P* < 0.001) (Table S16). No other biomarkers demonstrated consistent associations with HW exposure, highlighting the specificity of HW-related metabolic disruptions.

### Transcriptomic analysis, network construction, and functional enrichment

**Fig. 4.**
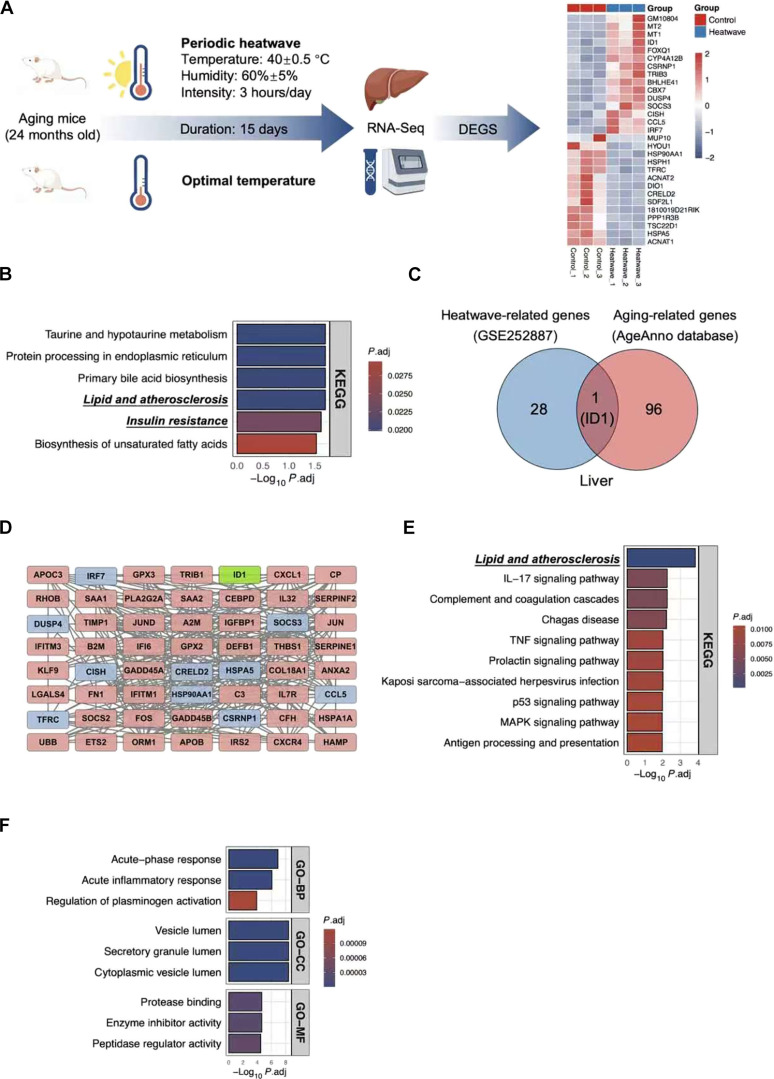
Transcriptomic and network analyses of HW-induced biological aging in aged mice. (A) Experimental design and differential gene expression analysis of liver tissues from aged mice (24 months old) subjected to periodic HW exposure for 15 d (GSE252887; *n* = 3 per group). (B) Kyoto Encyclopedia of Genes and Genomes (KEGG) pathway enrichment of HW-responsive DEGs, highlighting pathways involved in lipid metabolism and insulin resistance. (C) Venn diagram showing the intersection between HW-induced DEGs and aging-related genes curated from the AgeAnno database, based on single-cell RNA sequencing of human hepatocytes. (D) Key regulatory module identified using MCODE clustering; red nodes denote aging-related genes, blue nodes HW-responsive genes, and the green node (ID1) represents the shared gene. (E) KEGG enrichment of core module genes, highlighting lipid metabolism as the top enriched pathway. (F) Gene Ontology (GO) enrichment analysis of core module genes across biological processes (BP), cellular components (CC), and molecular functions (MF).

Transcriptomic profiling of liver tissues from aged mice identified 29 DEGs in response to HW exposure, including 15 up-regulated and 14 down-regulated genes (Fig. 4A). KEGG analysis showed enrichment in pathways related to “Lipid and Atherosclerosis” (mmu05417, adjusted *P* = 0.0198) and “Insulin Resistance” (mmu04931, adjusted *P* = 0.0239) (Fig. 4B). GO analysis further indicated involvement in protein folding-related processes, including “response to topologically incorrect protein” and “endoplasmic reticulum chaperone complex” (Fig. S5).

To assess aging-related relevance, HW-related DEGs were integrated with 97 aging-associated genes from the Aging Anno database (Fig. 4C), identifying ID1 as the only overlapping gene. The resulting PPI network comprised 116 nodes and 465 edges (*P* < 1.0 × 10^−16^) (Fig. S6). MCODE clustering identified a core module of 56 nodes and 300 edges with an average score of 10.90 (Fig. 4D).

Functional analysis of the core module showed significant enrichment in the “Lipid and Atherosclerosis” pathway (hsa05417, adjusted *P* < 0.001) (Fig. 4E). GO enrichment further highlighted processes including “acute-phase response” and “regulation of plasminogen activation”, along with vesicle-related components and protease-binding functions (Fig. 4F).

## Discussion

In this multi-scale study integrating population-based epidemiological analyses with experimental evidence, we found that HW exposure was associated with accelerated biological aging among middle-aged and older adults. These associations were more pronounced among individuals with higher BMI, those residing in urban areas, and those living in southern or subtropical regions of China, indicating potential effect heterogeneity across metabolic and environmental contexts. HW exposure was also consistently linked to metabolic dysregulation, particularly elevated TC and HbA1c levels. Complementary transcriptomic analyses of aged mice exposed to intermittent HW further identified DEGs enriched in pathways related to lipid metabolism and insulin resistance, providing biological plausibility for the observed epidemiological associations. Taken together, our findings suggest that HW exposure represents an important environmental stressor linked to accelerated biological aging, potentially mediated through metabolic dysregulation.

### HW exposure and aging-related health risks

As global warming intensifies, HWs are becoming increasingly frequent, prolonged, and severe, thereby amplifying their associated health burdens [28]. Accumulating epidemiological evidence suggests that HW exposure is associated with multiple aging-related health conditions. For example, Zhou et al. [29] reported an increased incidence of hypertension among older adults following HW events, while Yang et al. [30] observed an elevated risk of stroke, particularly among individuals with preexisting metabolic disorders such as diabetes or dyslipidemia. Cheng et al. [31] further demonstrated acute cardiovascular alterations—including elevations in blood pressure, heart rate, and systemic inflammation—after short-term HW exposure. Notably, these physiological disturbances mirror key hallmarks of biological aging, including vascular stiffening, metabolic dysregulation, and chronic low-grade inflammation. In this context, our findings extend previous research by suggesting that repeated HW exposure may be associated not only with transient stress responses but also with longer-term processes relevant to the progression of biological aging.

### Metabolic dysregulation as a potential mechanism

The consistent associations observed between HW exposure and elevated TC and HbA1c levels in our cohort suggest that disturbances in lipid and glucose metabolism may represent key biological pathways linking heat stress to accelerated biological aging. Because TC and HbA1c are components of the BA algorithm, these associations should be interpreted as supportive metabolic signatures rather than independent mechanistic mediators. HW-associated elevations in TC and HbA1c point to lipid and glucose metabolic disruption as a key pathway linking heat stress to biological aging. Deep learning-based body composition analysis now enables precision cardiometabolic risk profiling beyond conventional biomarkers [32], suggesting that artificial intelligence (AI)-driven quantification of metabolic phenotypes could help identify individuals most vulnerable to heat-induced accelerated aging. This hypothesis is further supported by transcriptomic profiling of aged mice exposed to intermittent HW, which identified 29 DEGs enriched in pathways related to lipid metabolism and insulin resistance. These pathways are biologically relevant to aging, as dysregulation of lipid handling and insulin signaling represents a central hallmark of systemic physiological decline. Heat stress can induce oxidative stress, mitochondrial dysfunction, and endoplasmic reticulum stress, thereby disrupting metabolic homeostasis and promoting chronic low-grade inflammation. Such processes are closely linked to established aging mechanisms, including impaired proteostasis, altered nutrient sensing, and increased cellular stress responses. Therefore, the transcriptomic alterations observed in heat-exposed aged mice provide mechanistic support for a pathway linking thermal stress to metabolic dysregulation and accelerated physiological aging. However, these findings should be interpreted as supportive experimental evidence rather than definitive mechanistic confirmation, and validation in human molecular or multi-omics studies is warranted. Given the limited sample size of the transcriptomic experiment, these findings should be considered exploratory and supportive rather than definitive mechanistic evidence. These molecular alterations align with previous findings in experimental models. For example, Song et al. [33] reported that ApoE^−/−^ mice exposed to HW exhibited persistent hyperlipidemia and myocardial injury, while Guo et al. [34] observed hepatic lipid dysregulation in heat-stressed chickens. Epidemiological evidence further supports a sustained impact of HW exposure on glucose metabolism. Xu et al. [35] found higher diabetes prevalence and increased hospitalizations among children following HW events, and Zheng et al. [36] documented elevated mortality from type 2 diabetes during HW periods in coastal Chinese cities. Together, these converging lines of evidence suggest that chronic metabolic dysregulation induced by heat stress may represent a plausible biological pathway linking environmental exposure to accelerated aging.

### Vulnerable subpopulations

Our findings highlight differential susceptibility to HW-induced biological aging across population subgroups. Individuals with elevated BMI may be particularly vulnerable due to impaired heat dissipation, increased cardiovascular load, and systemic inflammation [31]. Urban residents may also experience intensified thermal exposure owing to the urban heat island effect, which amplifies nocturnal and cumulative temperature stress. Socioeconomic differences may further modify vulnerability, as access to cooling resources, housing conditions, and health care can influence individual resilience to heat stress. Consistent with our observations, Tao et al. [37] found significantly higher HW-related diabetes mortality among urban compared with rural residents. Additionally, residents of southern and subtropical regions are exposed to greater baseline temperatures and more frequent HW events, potentially leading to cumulative physiological strain. These findings underscore the need for adaptive public health strategies that target vulnerable groups, integrating personalized and regional approaches to climate resilience. Such strategies may include targeted heat–health warning systems, urban heat mitigation measures, and community-level interventions aimed at reducing heat exposure among high-risk populations.

### Limitations

This study has several limitations that should be noted. First, although the DiD framework helps mitigate unmeasured confounding by relying on within-individual comparisons over time and thereby controlling for time-invariant individual characteristics, the observational design precludes definitive causal inference. In addition, the exposure window was limited to the 12 months preceding biomarker assessment, which may not fully capture cumulative lifetime thermal exposure relevant to biological aging processes. Moreover, HW exposure was estimated using city-level meteorological data, which may not fully reflect individual-level variability arising from microclimatic conditions or behavioral factors. This may have introduced nondifferential exposure misclassification, potentially biasing effect estimates toward the null. Third, the use of a Chinese cohort may limit the applicability of these results to populations with different demographic, genetic, socioeconomic, or climatic backgrounds. Fourth, BA was assessed using a validated clinical biomarker-based metric that primarily reflects systemic physiological aging rather than specific molecular aging processes. Comparisons with alternative biological aging metrics, such as molecular clocks or machine learning-based estimators, may provide complementary perspectives and should be explored in future studies. City-level meteorological data may misclassify individual exposure due to microclimatic and behavioral variability. Wearable technologies enabling real-time physiological monitoring offer solutions for personalized exposure assessment. Systems developed for space physiology applications demonstrate the feasibility of continuous monitoring under extreme thermal stress [38], and their integration into environmental epidemiology could improve exposure precision for heat-related aging research. Fifth, although baseline characteristics were largely comparable between included and excluded participants, the possibility of selection bias cannot be completely ruled out. Finally, although the overall sample size was substantial, statistical power may have been limited in certain subgroup analyses; these findings should therefore be interpreted as exploratory. Future studies incorporating individual-level exposure assessment, molecular aging biomarkers, and more diverse populations across different climatic regions will help refine understanding of the long-term biological impacts of heat exposure. Taken together, these limitations reflect inherent measurement uncertainty and constraints typical of observational study designs.

## Conclusion

Our findings indicate that HW exposure is linked to accelerated biological aging in middle-aged and older adults, particularly among individuals with higher BMI, urban residents, and those living in southern or subtropical regions. The observed increases in total cholesterol and glycated hemoglobin, together with transcriptomic signatures enriched in lipid metabolism and insulin resistance-related pathways, support metabolic dysregulation as a plausible mechanism underlying the aging-related impacts of heat stress. Collectively, these findings identify HWs as an important environmental stressor linked to biological aging and underscore the need for targeted strategies to enhance climate resilience and protect metabolically vulnerable populations. Although clinical endpoints were not evaluated in this study, BAA has been consistently associated with morbidity, functional decline, and mortality in prior research, supporting its relevance as an early indicator of health risk.

## Ethical Approval

Ethical approval was granted by Peking University (IRB00001052-11015), and written informed consent was obtained from all participants.

## Data Availability

Data will be made available on request.

## References

[1] Stewart CE, Sharples AP. Aging, skeletal muscle, and epigenetics. Plast Reconstr Surg. 2022;150:27S–33S. 36170433 10.1097/PRS.0000000000009670

[2] Campisi J, Kapahi P, Lithgow GJ, Melov S, Newman JC, Verdin E. From discoveries in ageing research to therapeutics for healthy ageing. Nature. 2019;571(7764):183–192. 31292558 10.1038/s41586-019-1365-2 PMC7205183

[3] Ferrucci L, Levine ME, Kuo PL, Simonsick EM. Time and the metrics of aging. Circ Res. 2018;123(7):740–744. 30355074 10.1161/CIRCRESAHA.118.312816 PMC6205734

[4] Klemera P, Doubal S. A new approach to the concept and computation of biological age. Mech Ageing Dev. 2006;127(3):240–248. 16318865 10.1016/j.mad.2005.10.004

[5] Lopez-Otin C, Blasco MA, Partridge L, Serrano M, Kroemer G. Hallmarks of aging: An expanding universe. Cell. 2023;186(2):243–278. 36599349 10.1016/j.cell.2022.11.001

[6] Kennedy BK, Berger SL, Brunet A, Campisi J, Cuervo AM, Epel ES, Franceschi C, Lithgow GJ, Morimoto RI, Pessin JE, et al. Geroscience: Linking aging to chronic disease. Cell. 2014;159(4):709–713. 25417146 10.1016/j.cell.2014.10.039 PMC4852871

[7] Rising J, Tedesco M, Piontek F, Stainforth DA. The missing risks of climate change. Nature. 2022;610(7933):643–651. 36289386 10.1038/s41586-022-05243-6

[8] The L. Heatwaves and health. Lancet. 2018;392(10145):359. 30102157 10.1016/S0140-6736(18)30434-3

[9] Xu R, Huang S, Shi C, Wang R, Liu T, Li Y, et al. Extreme temperature events, fine particulate matter, and myocardial infarction mortality. Circulation. 2023;148(4):312–323. 37486993 10.1161/CIRCULATIONAHA.122.063504

[10] Xi D, Liu L, Zhang M, Huang C, Burkart KG, Ebi K, Zeng Y, Ji JS. Risk factors associated with heatwave mortality in Chinese adults over 65 years. Nat Med. 2024;30(5):1489–1498. 38528168 10.1038/s41591-024-02880-4

[11] Chen J, Yang J, Zhou M, Yin P, Wang B, Liu J, Chen Z, Song X, Ou C-Q, Liu Q. Cold spell and mortality in 31 Chinese capital cities: Definitions, vulnerability and implications. Environ Int. 2019;128:271–278. 31071590 10.1016/j.envint.2019.04.049

[12] Ma C, Yang J, Nakayama SF, Iwai-Shimada M, Jung CR, Sun XL, Honda Y. Cold spells and cause-specific mortality in 47 Japanese prefectures: A systematic evaluation. Environ Health Perspect. 2021;129(6):67001. 34128690 10.1289/EHP7109 PMC8204943

[13] Jiang Y, Yi S, Gao C, Chen Y, Chen J, Fu X, Yang L, Kong X, Chen M, Kan H, et al. Cold spells and the onset of acute myocardial infarction: A nationwide case-crossover study in 323 Chinese cities. Environ Health Perspect. 2023;131(8):87016. 37610263 10.1289/EHP11841 PMC10445528

[14] Zhou W, Wang Q, Li R, Zhang Z, Wang W, Zhou F, Ling L. The effects of heatwave on cognitive impairment among older adults: Exploring the combined effects of air pollution and green space. Sci Total Environ. 2023;904: Article 166534. 37647952 10.1016/j.scitotenv.2023.166534

[15] Zhang X, Chen F, Chen Z. Heatwave and mental health. J Environ Manag. 2023;332: Article 117385. 10.1016/j.jenvman.2023.117385 36738719

[16] Alied M, Huy NT. A reminder to keep an eye on older people during heatwaves. Lancet Healthy Longev. 2022;3(10):e647–e648. 36202123 10.1016/S2666-7568(22)00198-2

[17] Li J, Li J, Wang X, Zhan X, Zeng Z. A domain generalization and residual network-based emotion recognition from physiological signals. Cyborg Bionic Syst. 2024;5:0074. 40391297 10.34133/cbsystems.0074 PMC12087918

[18] Zhao Y, Hu Y, Smith JP, Strauss J, Yang G. Cohort profile: The China Health and Retirement Longitudinal Study (CHARLS). Int J Epidemiol. 2014;43(1):61–68. 23243115 10.1093/ije/dys203 PMC3937970

[19] Chen X, Crimmins E, Hu PP, Kim JK, Meng Q, Strauss J, Wang Y, Zeng J, Zhang Y, Zhao Y. Venous blood-based biomarkers in the China Health and Retirement Longitudinal Study: Rationale, design, and results from the 2015 wave. Am J Epidemiol. 2019;188(11):1871–1877. 31364691 10.1093/aje/kwz170 PMC6825825

[20] Chen L, Wu B, Mo L, Chen H, Zhao Y, Tan T, Chen L, Li Y, Yao P, Tang Y. Associations between biological ageing and the risk of, genetic susceptibility to, and life expectancy associated with rheumatoid arthritis: A secondary analysis of two observational studies. Lancet Healthy Longev. 2024;5(1):e45–e55. 38081205 10.1016/S2666-7568(23)00220-9

[21] Yang Z, Shen Y, Zhang T, Tang X, Mao R. Associations of biological age accelerations and genetic risk with incident endometrial cancer: A prospective analysis in UK Biobank. Int J Surg. 2024;111(1):512–519. 10.1097/JS9.0000000000001966 PMC11745683 39017746

[22] Liu Z. Development and validation of 2 composite aging measures using routine clinical biomarkers in the Chinese population: Analyses from 2 prospective cohort studies. J Gerontol A Biol Sci Med Sci. 2021;76(9):1627–1632. 32946548 10.1093/gerona/glaa238 PMC8521780

[23] Jiang HP, Chen ZZ, Wang P, Li DB, Tao YC, Hong XL, Jiao X, Xia S, Zhang W. Association between biological age and contrast-associated acute kidney injury in patients undergoing coronary angiography a cross-sectional study. Cardiovasc Innov App. 2024;9(1):985.

[24] Lewinsohn PM, Seeley JR, Roberts RE, Allen NB. Center for Epidemiologic Studies Depression Scale (CES-D) as a screening instrument for depression among community-residing older adults. Psychol Aging. 1997;12(2):277–287. 9189988 10.1037//0882-7974.12.2.277

[25] Roy S, Saha P, Bose D, Trivedi A, More M, Lin C, Wu J, Oakes M, Chatterjee S. Periodic heat waves-induced neuronal etiology in the elderly is mediated by gut-liver-brain axis: A transcriptome profiling approach. Sci Rep. 2024;14(1):10555. 38719902 10.1038/s41598-024-60664-9 PMC11079080

[26] Xue T, Zhu T. Increment of ambient exposure to fine particles and the reduced human fertility rate in China, 2000-2010. Sci Total Environ. 2018;642:497–504. 29908508 10.1016/j.scitotenv.2018.06.075

[27] Xue T, Zhu T, Zheng Y, Zhang Q. Declines in mental health associated with air pollution and temperature variability in China. Nat Commun. 2019;10(1):2165. 31092825 10.1038/s41467-019-10196-y PMC6520357

[28] Shetty D. A lack of quality statistics is hiding the real heatwave death toll. BMJ. 2024;385: Article q1052. 38744464 10.1136/bmj.q1052

[29] Zhou W, Wang Q, Li R, Kadier A, Wang W, Zhou F, Ling L. Combined effects of heatwaves and air pollution, green space and blue space on the incidence of hypertension: A national cohort study. Sci Total Environ. 2023;867: Article 161560. 36640878 10.1016/j.scitotenv.2023.161560

[30] Yang C, Li Y, Huang C, Hou Y, Chu D, Bao J. Modification effects of immigration status and comorbidities on associations of heat and heatwave with stroke morbidity. Int J Stroke. 2024;19(9):1038–1045. 38863348 10.1177/17474930241263725

[31] Cheng BJ, Li H, Meng K, Li TL, Meng XC, Wang J, Wang C, Jiang N, Sun MJ, Yang LS, et al. Short-term effects of heatwaves on clinical and subclinical cardiovascular indicators in Chinese adults: A distributed lag analysis. Environ Int. 2024;183: Article 108358. 38056095 10.1016/j.envint.2023.108358

[32] Wei J, Chen H, Yao L, Hou X, Zhang R, Shi L, Sun J, Hu C, Wei X, Jia W. BioCompNet: A deep learning workflow enabling automated body composition analysis toward precision management of cardiometabolic disorders. Cybora Metabolic Dysrg Bionic Syst. 2025;6:0381. 10.34133/cbsystems.0381 PMC12367250 40842914

[33] Song QQ, Niu JP, Zhang SY, Liang TT, Zhou J, Feng SS. Effects of simulated heat wave and ozone on high fat diet ApoE deficient mice. Biomed Environ Sci. 2018;31(10):757–768. 30423277 10.3967/bes2018.101

[34] Guo Y, Liao JH, Liang ZL, Balasubramanian B, Liu WC. Hepatic lipid metabolomics in response to heat stress in local broiler chickens breed (Huaixiang chickens). Vet Med Sci. 2021;7(4):1369–1378. 33639042 10.1002/vms3.462 PMC8294384

[35] Xu Z, Tong S, Cheng J, Crooks JL, Xiang H, Li X, Huang C, Hu W. Heatwaves and diabetes in Brisbane, Australia: A population-based retrospective cohort study. Int J Epidemiol. 2019;48(4):1091–1100. 30927429 10.1093/ije/dyz048

[36] Zheng W, Chu J, Bambrick H, Wang N, Mengersen K, Guo X, Hu W. Impacts of heatwaves on type 2 diabetes mortality in China: A comparative analysis between coastal and inland cities. Int J Biometeorol. 2024;68(5):939–948. 38407634 10.1007/s00484-024-02638-0 PMC11058751

[37] Tao J, Zheng H, Ho HC, Wang X, Hossain MZ, Bai Z, Wang N, Su H, Xu Z, Cheng J. Urban-rural disparity in heatwave effects on diabetes mortality in eastern China: A case-crossover analysis in 2016-2019. Sci Total Environ. 2023;858(Pt 2): Article 160026. 36356755 10.1016/j.scitotenv.2022.160026

[38] Khan SUE, Varghese RJ, Kassanos P, Farina D, Burdet E. Space physiology and technology: Adaptations, countermeasures, and opportunities for wearable systems. Cyborg Bionic Syst. 2026;7:0477. 42004690 10.34133/cbsystems.0477 PMC13087406

