# Supplementary material for: Heatwave Exposure Accelerates Biological Aging via Metabolic Dysregulation
Source: Cyborg Bionic Syst. 2026 Jul 15;7:0602. doi: 10.34133/cbsystems.0602 (PMC13369309; doi:10.34133/cbsystems.0602)
Supplement: Supplementary 1 — Figs. S1 to S6 Tables S1 to S16 [file cbsystems.0602.f1.docx]

**Supplementary Materials**

**Figures**

1. Causal directed acyclic graph (DAG) depicting hypothesized pathways linking changes in heatwave and biological age acceleration.
2. Distribution of biological age and biological age acceleration in 2011 and 2015.
3. Correlation matrix among log-transformed biological age constituent biomarkers.
4. Association between baseline heatwave exposure and biological age acceleration.
5. Gene ontology enrichment analysis of heatwave-associated genes.
6. Protein–protein interaction (PPI) network of the combined gene set.

**Tables**

1. Baseline characteristics of included and excluded participants.
2. Baseline characteristics of participants in included and excluded cities.
3. City characteristics, climate zones, and sample sizes.
4. Definitions and population exposure rates of heatwave metrics.
5. Temporal changes in heatwave exposure between 2011 and 2015 across definitions.
6. Biological age and constituent biomarkers at baseline and follow-up.
7. Comparison of baseline and longitudinal characteristics between participants with accelerated and delayed biological aging.
8. Association between baseline heatwave exposure and biological age acceleration.
9. Longitudinal association between changes in heatwave exposure and changes in biological age acceleration across three models.
10. Longitudinal association between changes in heatwave exposure and accelerated biological aging across three models.
11. Associations between changes in heatwave exposure and biological age acceleration in a linear mixed-effects framework.
12. Association between changes in heatwave exposure and biological age acceleration after excluding potential outliers (|ΔBAA| > 10 year).
13. Association between changes in heatwave exposure and biological age acceleration after excluding potential outliers (|ΔBAA| > 5 year).
14. Association between changes in heatwave exposure and accelerated biological aging after excluding minimal change observations (|ΔBAA| < 1 year).
15. Statistical significance in associations between changes in heatwave exposure and changes in biological age–related biomarkers.
16. Association between changes in heatwave exposure and changes in HbA1c and total cholesterol.

**Figure S1.** Causal directed acyclic graph (DAG) depicting hypothesized pathways linking changes in heatwave and biological age acceleration.

**
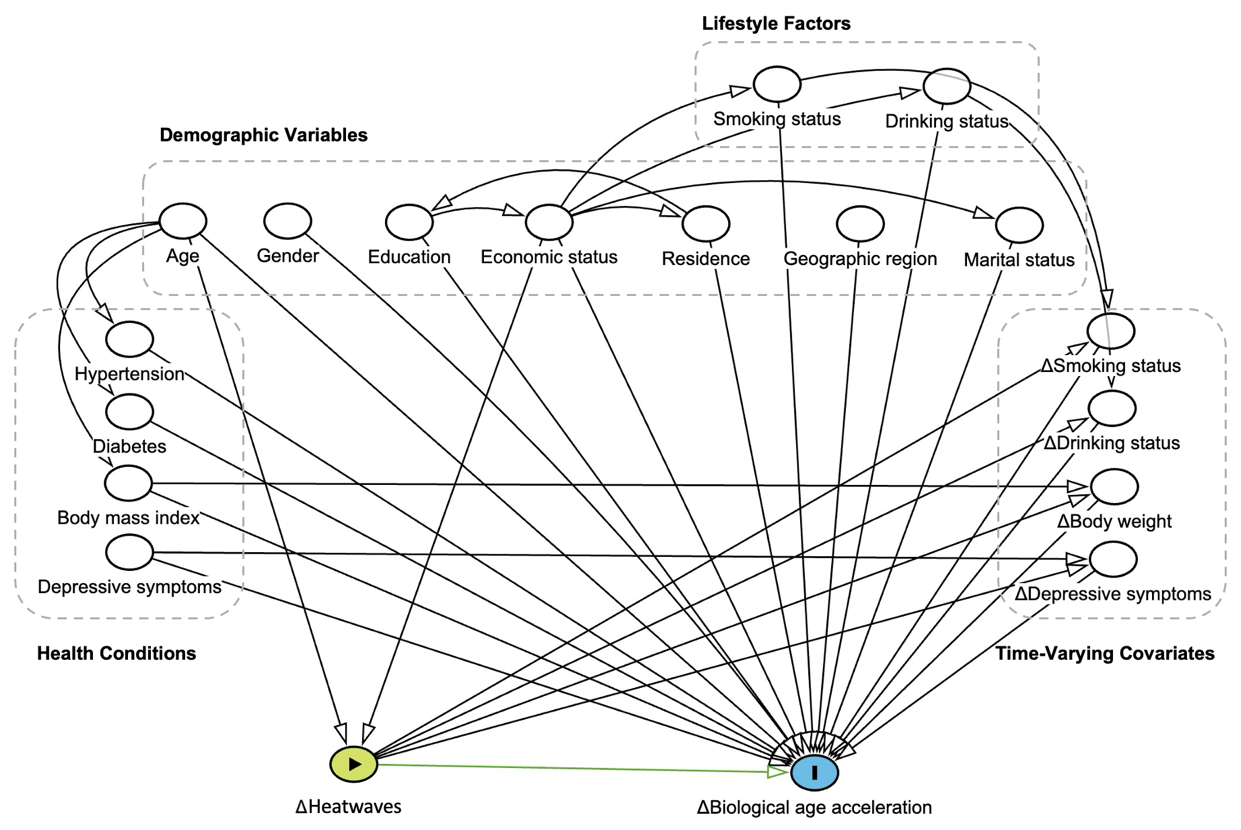
**

This directed acyclic graph (DAG) illustrates the hypothesized pathways linking changes in heatwave (HW) to biological age acceleration (BAA) while accounting for potential confounders and mediators across four domains: demographic variables, health conditions, lifestyle factors, and time-varying covariates.

**Figure S2.** Distribution of biological age and biological age acceleration in 2011 and 2015**.**

**
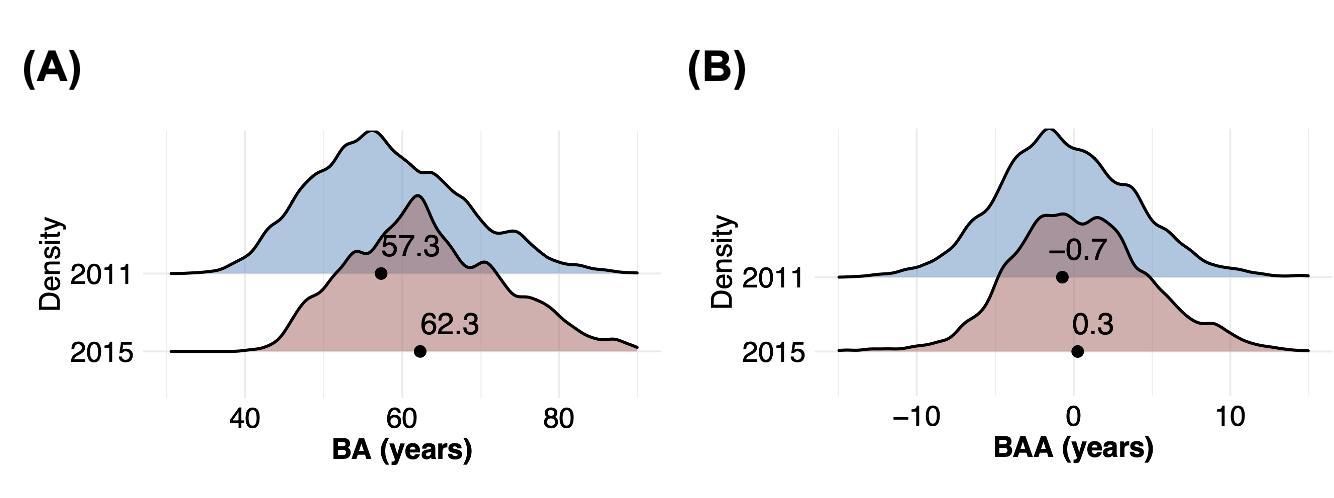
**

(A) Distribution of biological age (BA) in 2011 (blue) and 2015 (red); mean BA increased from 57.3 to 62.3 years. (B) Distribution of biological age acceleration (BAA); mean BAA shifted from –0.7 to 0.3 years over time.

**Figure S3.** Correlation matrix among log-transformed biological age constituent biomarkers.


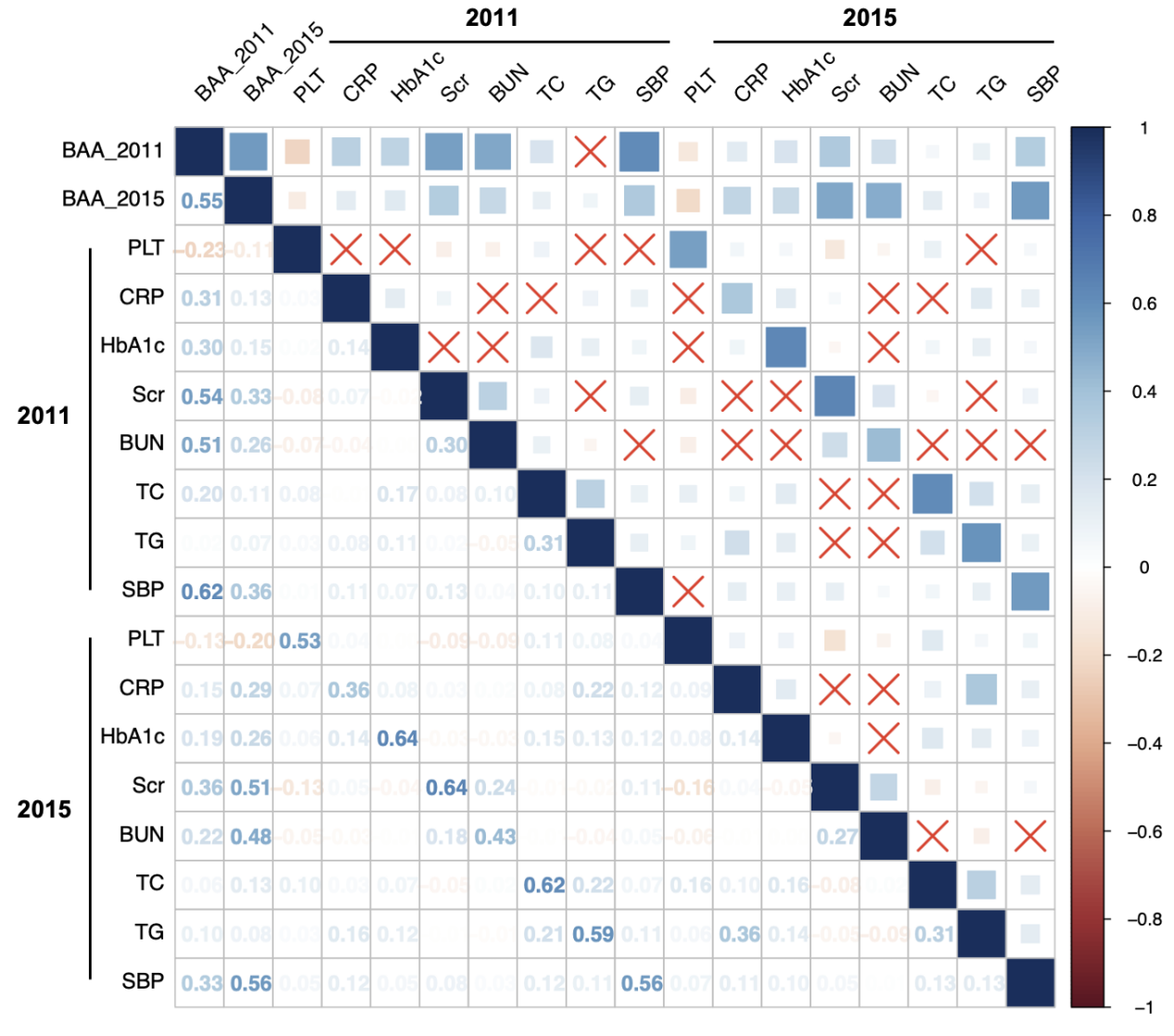


This figure presents the Pearson correlation coefficients among log-transformed biomarkers, including biological age acceleration (BAA), platelet count (PLT), C-reactive protein (CRP), glycated hemoglobin (HbA1c), serum creatinine (Scr), blood urea nitrogen (BUN), total cholesterol (TC), triglycerides (TG), and systolic blood pressure (SBP), measured in 2011 and 2015. The upper triangle displays the correlation coefficients, while the lower triangle provides their corresponding numerical values. Non-significant correlations (*p* > 0.05) are marked with red crosses.

**Figure S4.** Association between baseline heatwave exposure and biological age acceleration.


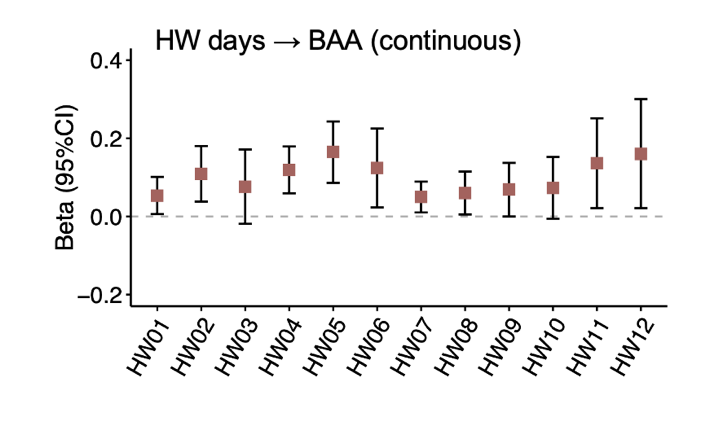


Beta coefficients (years per heatwave day) with 95% confidence intervals are presented across multiple heatwave (HW) definitions. Biological age acceleration (BAA) was defined as the difference between biological and chronological age. Heatwave exposure was quantified as the number of heatwave days during the 12 months prior to the baseline survey in 2011. Associations were estimated using generalized linear models adjusted for baseline covariates, including age, sex, body mass index, education, economic status, residence, geographic region, marital status, smoking, alcohol consumption, depressive symptoms, hypertension, and diabetes.

**Figure S5**. Gene ontology enrichment analysis of heatwave-associated genes.


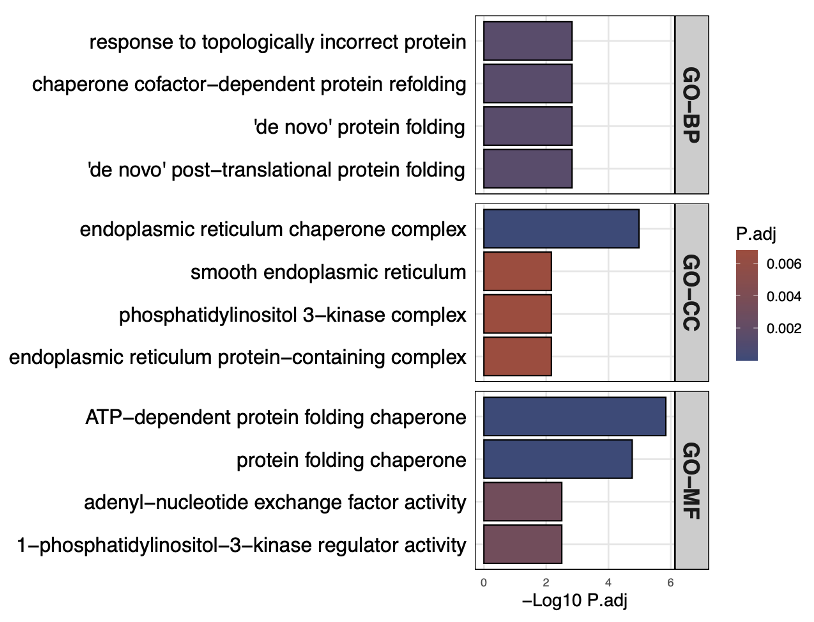


Gene Ontology (GO) enrichment analysis of genes targeted by heatwave (HW) is presented. The analysis identifies biological processes, molecular functions, and cellular components significantly enriched among HW-targeted genes. The bar plots show the top GO terms, with the y-axis representing the GO term categories and the x-axis indicating the significance level.

**Figure S6**. Protein–protein interaction (PPI) network of the combined gene set


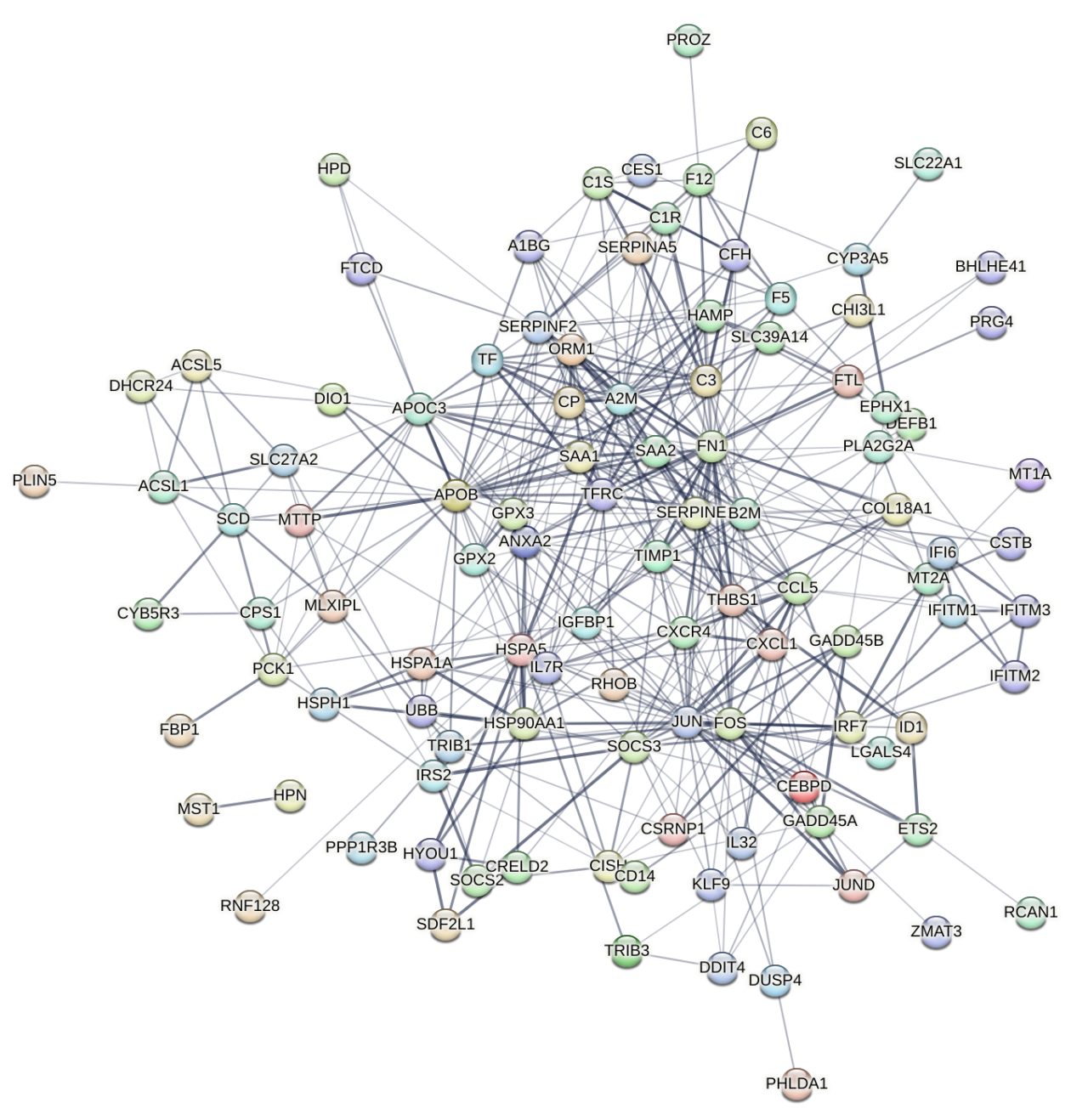


The edge thickness indicates interaction confidence.

**Table S1.** Baseline characteristics of included and excluded participants.

| ﻿Characteristics | | Participants interviewed both in 2011 & 2015 (n = 14574) | | SMD |
| --- | --- | --- | --- | --- |
|  |  | Included (n = 2318) | Excluded (n = 12256) |  |
| Age, years | | 58.67 ± 8.65 | 58.35 ± 9.66 | 0.035 |
| Gender, n (%) | |  |  |  |
|  | Female | 1231 (53.1) | 6461 (52.7) | 0.008 |
|  | Male | 1087 (46.9) | 5795 (47.3) |  |
| Body mass index, kg/m^2^ | | 23.41 ± 3.78 | 23.55 ± 3.87 | 0.037 |
| ﻿Education attainment, n (%) | |  |  |  |
|  | Illiterate | 634 (27.4) | 3341 (27.3) | 0.035 |
|  | Literate | 449 (19.4) | 2215 (18.1) |  |
|  | Elementary school and above | 1235 (53.3) | 6700 (54.7) |  |
| ﻿Income Status, n (%) | |  |  |  |
|  | No | 2049 (88.4) | 10020 (81.8) | 0.187 |
|  | Yes | 269 (11.6) | 2236 (18.2) |  |
| Residence, n (%) | |  |  |  |
|  | Rural | 1603 (69.2) | 7672 (62.6) | 0.139 |
|  | Urban | 715 (30.8) | 4584 (37.4) |  |
| Marital status, n (%) | |  |  |  |
|  | Married | 2068 (89.2) | 10817 (88.3) | 0.030 |
|  | Others | 250 (10.8) | 1439 (11.7) |  |
| ﻿Smoking status at baseline, n (%) | |  |  |  |
|  | Never | 1410 (60.8) | 7506 (61.2) | 0.073 |
|  | Quit | 207 (8.9) | 1323 (10.8) |  |
|  | Current | 701 (30.2) | 3427 (28.0) |  |
| Drinking status at baseline, n (%) | |  |  |  |
|  | Never | 1494 (64.5) | 8241 (67.2) | 0.060 |
|  | Less than once a month | 637 (27.5) | 3066 (25.0) |  |
|  | More than once a month | 187 (8.1) | 949 (7.7) |  |
| ﻿Self-reported hypertension, n (%) | |  |  |  |
|  | No | 1753 (75.6) | 9424 (76.9) | 0.030 |
|  | Yes | 565 (24.4) | 2832 (23.1) |  |
| Self-reported ﻿diabetes, n (%) | |  |  |  |
|  | No | 2208 (95.3) | 11596 (94.6) | 0.029 |
|  | Yes | 110 (4.7) | 660 (5.4) |  |

Baseline characteristics of participants interviewed in both 2011 and 2015 were compared between those included in the analysis (n = 2,318) and those excluded (n = 12,256). Continuous variables are presented as mean ± standard deviation (SD), while categorical variables are expressed as count (percentage). Standardized mean differences (SMD) are provided for each characteristic to indicate the magnitude of differences between groups.

**Table S2.** Baseline characteristics of participants in included and excluded cities.

| ﻿Characteristics | | Participants in Included Cities  (n = 2318) | Participants in Excluded Cities  (n = 3505) | SMD |
| --- | --- | --- | --- | --- |
| Age, years | | 58.67 ± 8.65 | 59.18 ± 8.88 | 0.058 |
| Gender, n (%) | |  |  |  |
|  | Female | 1231 (53.1) | 1913 (54.6) | 0.030 |
|  | Male | 1087 (46.9) | 1592 (45.4) |  |
| Body Mass Index, kg/m2 | | 23.41 ± 3.78 | 23.82 ± 3.87 | 0.108 |
| ﻿Education attainment, n (%) | |  |  |  |
|  | Illiterate | 634 (27.4) | 1016 (29.0) | 0.038 |
|  | Literate | 449 (19.4) | 649 (18.5) |  |
|  | Elementary school and above | 1235 (53.3) | 1840 (52.5) |  |
| ﻿Income Status, n (%) | |  |  |  |
|  | No | 2049 (88.4) | 3003 (85.7) | 0.081 |
|  | Yes | 269 (11.6) | 502 (14.3) |  |
| Residence, n (%) | |  |  |  |
|  | Rural | 1603 (69.2) | 2320 (66.2) | 0.063 |
|  | Urban | 715 (30.8) | 1185 (33.8) |  |
| ﻿Geographic region, n (%) | |  |  |  |
|  | Northern China | 984 (42.5) | 1454 (41.5) | 0.020 |
|  | Southern China | 1334 (57.5) | 2051 (58.5) |  |
| Marital status, n (%) | |  |  |  |
|  | Married | 2068 (89.2) | 3105 (88.6) | 0.020 |
|  | Others | 250 (10.8) | 400 (11.4) |  |
| ﻿Smoking status at baseline, n (%) | |  |  |  |
|  | Never | 1410 (60.8) | 2169 (61.9) | 0.022 |
|  | Quit | 207 (8.9) | 302 (8.6) |  |
|  | Current | 701 (30.2) | 1034 (29.5) |  |
| Drinking status at baseline, n (%) | |  |  |  |
|  | Never | 1494 (64.5) | 2404 (68.6) | 0.093 |
|  | Less than once a month | 637 (27.5) | 825 (23.5) |  |
|  | More than once a month | 187 (8.1) | 276 (7.9) |  |
| ﻿Self-reported hypertension, n (%) | |  |  |  |
|  | No | 1753 (75.6) | 2611 (74.5) | 0.026 |
|  | Yes | 565 (24.4) | 894 (25.5) |  |
| Self-reported ﻿diabetes, n (%) | |  |  |  |
|  | No | 2208 (95.3) | 3286 (93.8) | 0.066 |
|  | Yes | 110 (4.7) | 219 (6.2) |  |
| ﻿Depression ﻿symptom score | | 8.00 [4.00, 13.00] | 7.00 [3.00, 12.00] | 0.094 |
| Platelet count, 10×9/L | | 207.00 [164.00, 257.00] | 207.00 [161.00, 255.00] | 0.061 |
| C-reactive protein, mg/L | | 0.99 [0.55, 2.07] | 1.02 [0.54, 2.12] | 0.015 |
| Hemoglobin A1c, % | | 5.10 [4.90, 5.40] | 5.20 [4.90, 5.40] | 0.079 |
| Serum creatinine, mg/dL | | 0.76 [0.66, 0.89] | 0.75 [0.64, 0.86] | 0.135 |
| Blood urea nitrogen, mg/dL | | 15.49 [12.92, 18.60] | 14.90 [12.46, 17.84] | 0.125 |
| Total cholesterol, mg/dL | | 190.21 [167.40, 214.95] | 190.59 [166.62, 214.95] | 0.002 |
| Triglycerides, mg/dL | | 103.54 [73.46, 152.22] | 107.08 [76.11, 157.53] | 0.009 |
| Systolic blood pressure, mmHg | | 126.33 [114.33, 141.67] | 127.67 [114.33, 142.00] | 0.026 |

Baseline characteristics of participants from 50 included cities (n = 2,318) and those from the remaining excluded cities (n = 3,505) were compared. Continuous variables are presented as mean ± standard deviation (SD) or median [interquartile range (IQR)], while categorical variables are expressed as count (percentage). Standardized mean differences (SMD) are provided for each characteristic to assess the magnitude of differences between groups.

**Table S3.** City characteristics, climate zones, and sample sizes.

| City Name  (Chinese) | City Name  (English) | Location | Climate Zone | Sample Count |
| --- | --- | --- | --- | --- |
| 保定市 | Baoding | Northern China | Temperate Monsoon Zone | 162 |
| 潍坊市 | Weifang | Northern China | Temperate Monsoon Zone | 104 |
| 信阳市 | Xinyang | Northern China | Subtropical Monsoon Zone | 91 |
| 张掖市 | Zhangye | Northern China | Temperate Continental Zone | 90 |
| 济南市 | Jinan | Northern China | Temperate Monsoon Zone | 83 |
| 四平市 | Siping | Northern China | Temperate Monsoon Zone | 77 |
| 朝阳市 | Chaoyang | Northern China | Temperate Monsoon Zone | 58 |
| 阜阳市 | Fuyang | Northern China | Temperate Monsoon Zone | 54 |
| 锦州市 | Jinzhou | Northern China | Temperate Monsoon Zone | 51 |
| 赤峰市 | Chifeng | Northern China | Temperate Monsoon Zone | 38 |
| 承德市 | Chengde | Northern China | Temperate Monsoon Zone | 32 |
| 榆林市 | Yulin | Northern China | Temperate Continental Zone | 30 |
| 鸡西市 | Jixi | Northern China | Temperate Monsoon Zone | 28 |
| 运城市 | Yuncheng | Northern China | Temperate Continental Zone | 23 |
| 锡林郭勒盟 | Xilingol | Northern China | Temperate Continental Zone | 23 |
| 平凉市 | Pingliang | Northern China | Temperate Continental Zone | 19 |
| 松原市 | Songyuan | Northern China | Temperate Monsoon Zone | 8 |
| 通辽市 | Tongliao | Northern China | Temperate Monsoon Zone | 6 |
| 徐州市 | Xuzhou | Northern China | Temperate Monsoon Zone | 5 |
| 张家口市 | Zhangjiakou | Northern China | Temperate Monsoon Zone | 2 |
| 楚雄彝族自治州 | Chuxiong | Southern China | Plateau Mountain Climate Zone | 117 |
| 邵阳市 | Shaoyang | Southern China | Plateau Mountain Climate Zone | 101 |
| 宜宾市 | Yibin | Southern China | Plateau Mountain Climate Zone | 92 |
| 安阳市 | Anyang | Southern China | Temperate Monsoon Zone | 77 |
| 玉林市 | Yulin | Southern China | Plateau Mountain Climate Zone | 76 |
| 赣州市 | Ganzhou | Southern China | Plateau Mountain Climate Zone | 60 |
| 绵阳市 | Mianyang | Southern China | Plateau Mountain Climate Zone | 58 |
| 保山市 | Baoshan | Southern China | Plateau Mountain Climate Zone | 53 |
| 成都市 | Chengdu | Southern China | Plateau Mountain Climate Zone | 53 |
| 常德市 | Changde | Southern China | Subtropical Monsoon Zone | 52 |
| 汉中市 | Hanzhong | Southern China | Plateau Mountain Climate Zone | 52 |
| 丽水市 | Lishui | Southern China | Plateau Mountain Climate Zone | 46 |
| 扬州市 | Yangzhou | Southern China | Subtropical Monsoon Zone | 46 |
| 临沧市 | Lincang | Southern China | Plateau Mountain Climate Zone | 43 |
| 昭通市 | Zhaotong | Southern China | Plateau Mountain Climate Zone | 43 |
| 襄阳市 | Xiangyang | Southern China | Subtropical Monsoon Zone | 43 |
| 安庆市 | Anqing | Southern China | Subtropical Monsoon Zone | 42 |
| 河池市 | Hechi | Southern China | Plateau Mountain Climate Zone | 40 |
| 恩施土家族苗族自治州 | Enshi | Southern China | Plateau Mountain Climate Zone | 38 |
| 长沙市 | Changsha | Southern China | Subtropical Monsoon Zone | 35 |
| 宜春市 | Yichun | Southern China | Subtropical Monsoon Zone | 33 |
| 盐城市 | Yancheng | Southern China | Subtropical Monsoon Zone | 25 |
| 福州市 | Fuzhou | Southern China | Subtropical Monsoon Zone | 22 |
| 景德镇市 | Jingdezhen | Southern China | Subtropical Monsoon Zone | 19 |
| 芜湖市 | Wuhu | Southern China | Subtropical Monsoon Zone | 17 |
| 岳阳市 | Yueyang | Southern China | Subtropical Monsoon Zone | 14 |
| 清远市 | Qingyuan | Southern China | Subtropical Monsoon Zone | 13 |
| 郑州市 | Zhengzhou | Southern China | Temperate Monsoon Zone | 11 |
| 吉安市 | Ji'an | Southern China | Subtropical Monsoon Zone | 9 |
| 杭州市 | Hangzhou | Southern China | Subtropical Monsoon Zone | 4 |

This table lists the Chinese and English names of each city, their geographical location, climate zone, and the corresponding sample size.

**Table S4.** ﻿Definitions and population exposure rates of heatwave metrics.

| HW Code | Definition | Percentile Threshold | ﻿Min Duration (Days) | % Exposed (2011) | % Exposed (2015) |
| --- | --- | --- | --- | --- | --- |
| HW1 | ﻿P90_2d | 90.0th | ≥ 2 | 82.2 | 77.7 |
| HW2 | P90_3d | 90.0th | ≥ 3 | 81 | 72.5 |
| HW3 | P90_4d | 90.0th | ≥ 4 | 74.8 | 61.3 |
| HW4 | P92.5_2d | 92.5th | ≥ 2 | 75.5 | 71.4 |
| HW5 | P92.5_3d | 92.5th | ≥ 3 | 68.7 | 63.8 |
| HW6 | P92.5_4d | 92.5th | ≥ 4 | 63.9 | 61.3 |
| HW7 | P95_2d | 95.0th | ≥ 2 | 61.4 | 63.8 |
| HW8 | P95_3d | 95.0th | ≥ 3 | 61.4 | 57 |
| HW9 | P95_4d | 95.0th | ≥ 4 | 61.3 | 52.5 |
| HW10 | P97.5_2d | 97.5th | ≥ 2 | 59.7 | 58.5 |
| HW11 | P97.5_3d | 97.5th | ≥ 3 | 59.7 | 38.2 |
| HW12 | P97.5_4d | 97.5th | ≥ 4 | 49.7 | 36.6 |

Summary of the twelve heatwave (HW) definitions used in the study, based on varying combinations of temperature percentile thresholds and minimum duration criteria. For each definition, the proportion of participants exposed in the baseline (2011) and follow-up (2015) waves of the China Health and Retirement Longitudinal Study (CHARLS) is reported. Temperature thresholds are defined based on city-specific daily maximum temperature percentiles, and exposure was assessed over the 12 months preceding each survey wave.

**Table S5.** Temporal changes in heatwave exposure between 2011 and 2015 across definitions.

| HW Code | Definition | △HWs, mean ± SD | |  | △HWs, Median [IQR] | |
| --- | --- | --- | --- | --- | --- | --- |
|  |  | Event counts | Day counts |  | Event counts | Day counts |
| HW1 | ﻿P90_2d | -1.4±3.2 | -10.49±11.7 |  | -1 [-4, 0] | -8 [-18, 0] |
| HW2 | P90_3d | -0.7±2.3 | -9.08±10.7 |  | -1 [-2, 0] | -6 [-14, -1] |
| HW3 | P90_4d | -0.26±1.6 | -7.76±11.1 |  | 0 [-1, 0] | -4 [-11, 0] |
| HW4 | P92.5_2d | -1.09±2 | -7.75±10 |  | -1 [-2, 0] | -3 [-17, 0] |
| HW5 | P92.5_3d | -0.52±1.7 | -6.62±9.8 |  | 0 [-1, 0] | -3 [-15, 0] |
| HW6 | P92.5_4d | -0.46±1.1 | -6.43±10 |  | 0 [-1, 0] | -6 [-12, 0] |
| HW7 | P95_2d | -0.87±1.8 | -5.38±10.5 |  | 0 [-2, 0] | 0 [-13, 0] |
| HW8 | P95_3d | -0.69±1.5 | -5.02±10.4 |  | 0 [-2, 0] | 0 [-12, 0] |
| HW9 | P95_4d | -0.74±1.2 | -5.16±10 |  | 0 [-1, 0] | 0 [-11, 0] |
| HW10 | P97.5_2d | -0.62±1.5 | -3.38±9.6 |  | 0 [-1, 0] | -3 [-6, 0] |
| HW11 | P97.5_3d | -0.54±1.1 | -3.23±8.8 |  | 0 [-1, 0] | -3 [-6, 0] |
| HW12 | P97.5_4d | -0.36±0.9 | -2.68±8.9 |  | 0 [-1, 0] | 0 [-7, 0] |

Summary of changes in heatwave (HW) exposure from baseline (2011) to follow-up (2015), based on twelve HW definitions combining temperature percentile thresholds and minimum duration criteria. Changes in HW exposure (ΔHWs) are presented as mean ± standard deviation (SD) and median with interquartile range [IQR], for both the number of HW events and total HW days. HW event counts refer to the frequency of distinct heatwave episodes, while day counts represent the cumulative duration of all HW events over the year preceding each survey wave.

**Table S6.** Biological age and constituent biomarkers at baseline and follow-up.

|  |  | Baseline (2011) | Follow-up (2015) |
| --- | --- | --- | --- |
| BAA, years | | -0.72 [-3.42, 2.38] | 0.25 [-2.52, 3.19] |
| Chronological age, years | | 58.7 ± 8.6 | 62.7 ± 8.6 |
| Biological age, years | | 58.2 ± 9.5 | 63.1 ± 9.6 |
| Components of biological age | |  |  |
|  | Platelet count, 10^9^/L | 215.77 ± 84.76 | 203.83 ± 71.08 |
|  | C-reactive protein, mg/L | 0.99 [0.55, 2.07] | 1.40 [0.80, 2.70] |
|  | Hemoglobin A1c, % | 5.23 ± 0.78 | 5.97 ± 0.91 |
|  | Serum creatinine, mg/dL | 0.79 ± 0.20 | 0.83 ± 0.33 |
|  | Blood urea nitrogen, mg/dL | 16.00 ± 4.49 | 16.06 ± 4.79 |
|  | Total cholesterol, mg/dL | 193.30 ± 37.46 | 186.00 ± 36.50 |
|  | Triglycerides, mg/dL | 134.80 ± 119.57 | 145.62 ± 96.69 |
|  | Systolic blood pressure, mmHg | 129.69 ± 21.45 | 129.78 ± 21.60 |

Summary of biological age acceleration (BAA), chronological age, biological age, and component biomarkers at baseline (2011) and follow-up (2015). BAA was defined as the difference between biological and chronological age, with positive values indicating accelerated aging. Continuous variables are presented as mean ± standard deviation (SD) or median [interquartile range, IQR], depending on distribution.

**Table S7.** Comparison of baseline and longitudinal characteristics between participants with accelerated and delayed biological aging.

| ﻿Characteristics | |  | Accelerated  (ΔBAA ≥ 0, n = 1363) | Delayed  (ΔBAA < 0, n = 955) | *p* value |
| --- | --- | --- | --- | --- | --- |
| Baseline ﻿characteristics (2011) | | |  |  |  |
|  | Age, years |  | 58.80 (8.73) | 58.48 (8.54) | 0.379 |
|  | Gender, n (%) |  |  |  |  |
|  |  | Female | 726 (53.3) | 505 (52.9) | 0.888 |
|  |  | Male | 637 (46.7) | 450 (47.1) |  |
|  | Body Mass Index, kg/m^2^ | | 23.36 (3.82) | 23.47 (3.73) | 0.498 |
|  | ﻿Education Attainment, n (%) | |  |  |  |
|  |  | Illiterate | 367 (26.9) | 267 (28.0) | 0.795 |
|  |  | Literate | 262 (19.2) | 187 (19.6) |  |
|  |  | Elementary school and above | 734 (53.9) | 501 (52.5) |  |
|  | ﻿Income Status, n (%) | |  |  |  |
|  |  | No | 1195 (87.7) | 854 (89.4) | 0.219 |
|  |  | Yes | 168 (12.3) | 101 (10.6) |  |
|  | Residence, n (%) | |  |  |  |
|  |  | Rural | 936 (68.7) | 667 (69.8) | 0.579 |
|  |  | Urban | 427 (31.3) | 288 (30.2) |  |
|  | ﻿Geographic Region, n (%) | |  |  |  |
|  |  | Northern China | 626 (45.9) | 358 (37.5) | <0.001 |
|  |  | Southern China | 737 (54.1) | 597 (62.5) |  |
|  | Marital status, n (%) | |  |  |  |
|  |  | Married | 1221 (89.6) | 847 (88.7) | 0.540 |
|  |  | Others | 142 (10.4) | 108 (11.3) |  |
|  | ﻿Smoking Status, n (%) | |  |  |  |
|  |  | Never | 854 (62.7) | 556 (58.2) | 0.088 |
|  |  | Quit | 119 (8.7) | 88 (9.2) |  |
|  |  | Current | 390 (28.6) | 311 (32.6) |  |
|  | Drinking Status, n (%) | |  |  |  |
|  |  | Never | 874 (64.1) | 620 (64.9) | 0.645 |
|  |  | Less than once a month | 373 (27.4) | 264 (27.6) |  |
|  |  | More than once a month | 116 (8.5) | 71 (7.4) |  |
|  | ﻿Self-Reported Hypertension, n (%) | |  |  |  |
|  |  | No | 1040 (76.3) | 713 (74.7) | 0.391 |
|  |  | Yes | 323 (23.7) | 242 (25.3) |  |
|  | Self-Reported ﻿Diabetes, n (%) | |  |  |  |
|  |  | No | 1303 (95.6) | 905 (94.8) | 0.407 |
|  |  | Yes | 60 (4.4) | 50 (5.2) |  |
|  | ﻿Depressive Symptoms (CESD-10) | | 8.00 [4.00, 13.00] | 8.00 [4.00, 13.00] | 0.741 |
| Changes in Characteristics (2015 vs. 2011) | | |  |  |  |
|  | Depressive Symptoms Change | | 0.00 [-4.00, 3.00] | 0.00 [-4.00, 3.50] | 0.858 |
|  | Drinking Status, n (%) | |  |  |  |
|  |  | No Change |  |  |  |
|  |  | From No to Yes | 1148 (84.2) | 790 (82.7) | 0.062 |
|  |  | From Yes to No | 115 (8.4) | 70 (7.3) |  |
|  | Smoking Status, n (%) | | 100 (7.3) | 95 (9.9) |  |
|  |  | No Change | 1265 (92.8) | 874 (91.5) | 0.478 |
|  |  | From No to Yes | 23 (1.7) | 17 (1.8) |  |
|  |  | From Yes to No | 75 (5.5) | 64 (6.7) |  |
|  | Weight Change (≥ 5kg), n (%) | |  |  |  |
|  |  | No Change | 1139 (83.6) | 806 (84.4) | 0.020 |
|  |  | Weight Gain | 142 (10.4) | 73 (7.6) |  |
|  |  | Weight Loss | 82 (6.0) | 76 (8.0) |  |

Baseline (2011) and follow-up changes (2015 vs. 2011) in demographic, behavioral, and clinical characteristics among participants with accelerated aging (ΔBAA ≥ 0, n = 1,363) and delayed aging (ΔBAA < 0, n = 955). Continuous variables are reported as mean (standard deviation) or median [interquartile range], and categorical variables as count (percentage). Between-group comparisons were performed using t-tests or Wilcoxon rank-sum tests for continuous variables and chi-square tests for categorical variables. ΔBAA was defined as the change in biological age relative to chronological age between baseline and follow-up.

**Table S8.** Association between baseline heatwave exposure and biological age acceleration.

| HW code | Beta | 95%CI | *p* value | FDR-adjusted *p* value |
| --- | --- | --- | --- | --- |
| HW01 | 0.053 | (0.006, 0.101) | 0.027 | 0.040 |
| HW02 | 0.109 | (0.038, 0.180) | 0.003 | 0.012 |
| HW03 | 0.076 | (-0.019, 0.171) | 0.117 | 0.117 |
| HW04 | 0.119 | (0.059, 0.179) | <0.001 | <0.001 |
| HW05 | 0.165 | (0.086, 0.243) | <0.001 | <0.001 |
| HW06 | 0.124 | (0.023, 0.225) | 0.016 | 0.038 |
| HW07 | 0.050 | (0.010, 0.089) | 0.014 | 0.038 |
| HW08 | 0.060 | (0.005, 0.115) | 0.032 | 0.043 |
| HW09 | 0.069 | (0.000, 0.137) | 0.049 | 0.059 |
| HW10 | 0.073 | (-0.006, 0.152) | 0.070 | 0.076 |
| HW11 | 0.136 | (0.021, 0.251) | 0.021 | 0.040 |

This table shows the associations between baseline heatwave (HW) days and biological age acceleration (BAA) at baseline. HW exposure was defined using multiple threshold-duration criteria. BAA was calculated as the difference between biological and chronological age. Estimates were derived from generalized linear models adjusted for age, sex, body mass index, education, income, residence, geographic region, marital status, smoking, alcohol consumption, depressive symptoms, hypertension, and diabetes. Beta coefficients indicate the change in BAA (years) per additional HW day. Both unadjusted and false discovery rate (FDR)–adjusted p values are reported to account for multiple comparisons.

**Table S9.** Longitudinal association between changes in heatwave exposure and changes in biological age acceleration across three models.

| HW code | | Model 1 (unadjusted) | | | |  | Model 2 (baseline covariates adjusted) | | | |  | Model 3 (baseline + time-varying covariates adjusted) | | | |
| --- | --- | --- | --- | --- | --- | --- | --- | --- | --- | --- | --- | --- | --- | --- | --- |
|  |  | Beta | 95%CI | *p* value | FDR-adjusted  *p* value |  | Beta | 95%CI | *p* value | FDR-adjusted  *p* value |  | Beta | 95%CI | *p* value | FDR-adjusted  *p* value |
| HW events | |  |  |  |  |  |  |  |  |  |  |  |  |  |  |
|  | HW01 | -0.008 | (-0.062, 0.045) | 0.767 | 0.767 |  | -0.004 | (-0.059, 0.052) | 0.892 | 0.892 |  | 0.001 | (-0.054, 0.056) | 0.973 | 0.973 |
|  | HW02 | 0.031 | (-0.044, 0.106) | 0.414 | 0.497 |  | 0.019 | (-0.057, 0.096) | 0.620 | 0.676 |  | 0.024 | (-0.053, 0.100) | 0.545 | 0.595 |
|  | HW03 | 0.121 | (0.014, 0.227) | 0.027 | 0.036 |  | 0.104 | (-0.006, 0.213) | 0.064 | 0.077 |  | 0.101 | (-0.008, 0.211) | 0.070 | 0.084 |
|  | HW04 | 0.156 | (0.072, 0.241) | <0.001 | <0.001 |  | 0.180 | (0.092, 0.267) | <0.001 | <0.001 |  | 0.179 | (0.091, 0.266) | <0.001 | <0.001 |
|  | HW05 | 0.346 | (0.246, 0.446) | <0.001 | <0.001 |  | 0.360 | (0.258, 0.462) | <0.001 | <0.001 |  | 0.363 | (0.261, 0.466) | <0.001 | <0.001 |
|  | HW06 | 0.207 | (0.056, 0.358) | 0.007 | 0.014 |  | 0.291 | (0.134, 0.449) | <0.001 | <0.001 |  | 0.295 | (0.137, 0.452) | <0.001 | <0.001 |
|  | HW07 | 0.112 | (0.017, 0.206) | 0.021 | 0.036 |  | 0.135 | (0.033, 0.238) | 0.010 | 0.015 |  | 0.135 | (0.032, 0.238) | 0.010 | 0.015 |
|  | HW08 | 0.131 | (0.016, 0.245) | 0.026 | 0.036 |  | 0.133 | (0.014, 0.252) | 0.028 | 0.037 |  | 0.138 | (0.02, 0.257) | 0.022 | 0.029 |
|  | HW09 | 0.043 | (-0.094, 0.181) | 0.540 | 0.589 |  | 0.135 | (0.033, 0.238) | 0.010 | 0.015 |  | 0.135 | (0.032, 0.238) | 0.010 | 0.015 |
|  | HW10 | 0.195 | (0.084, 0.306) | 0.001 | 0.002 |  | 0.212 | (0.095, 0.329) | <0.001 | <0.001 |  | 0.220 | (0.103, 0.337) | <0.001 | <0.001 |
|  | HW11 | 0.372 | (0.221, 0.523) | <0.001 | <0.001 |  | 0.420 | (0.258, 0.582) | <0.001 | <0.001 |  | 0.436 | (0.274, 0.597) | <0.001 | <0.001 |
|  | HW12 | 0.481 | (0.296, 0.667) | <0.001 | <0.001 |  | 0.525 | (0.334, 0.715) | <0.001 | <0.001 |  | 0.531 | (0.341, 0.722) | <0.001 | <0.001 |
| HW days | |  |  |  |  |  |  |  |  |  |  |  |  |  |  |
|  | HW01 | 0.010 | (-0.005, 0.025) | 0.187 | 0.204 |  | 0.016 | (0, 0.033) | 0.046 | 0.050 |  | 0.017 | (0.001, 0.033) | 0.038 | 0.041 |
|  | HW02 | 0.016 | (0, 0.032) | 0.049 | 0.065 |  | 0.023 | (0.005, 0.04) | 0.012 | 0.016 |  | 0.023 | (0.005, 0.040) | 0.011 | 0.015 |
|  | HW03 | 0.019 | (0.003, 0.034) | 0.018 | 0.027 |  | 0.025 | (0.008, 0.041) | 0.004 | 0.007 |  | 0.024 | (0.007, 0.041) | 0.005 | 0.007 |
|  | HW04 | 0.024 | (0.007, 0.041) | 0.006 | 0.014 |  | 0.034 | (0.016, 0.053) | <0.001 | <0.001 |  | 0.035 | (0.016, 0.053) | <0.001 | <0.001 |
|  | HW05 | 0.032 | (0.015, 0.050) | <0.001 | <0.001 |  | 0.041 | (0.023, 0.060) | <0.001 | <0.001 |  | 0.042 | (0.023, 0.060) | <0.001 | <0.001 |
|  | HW06 | 0.009 | (-0.008, 0.026) | 0.304 | 0.304 |  | 0.017 | (-0.001, 0.036) | 0.064 | 0.064 |  | 0.018 | (-0.001, 0.036) | 0.059 | 0.059 |
|  | HW07 | 0.022 | (0.005, 0.038) | 0.009 | 0.018 |  | 0.026 | (0.009, 0.044) | 0.002 | 0.004 |  | 0.027 | (0.010, 0.044) | 0.002 | 0.004 |
|  | HW08 | 0.021 | (0.004, 0.037) | 0.014 | 0.024 |  | 0.024 | (0.007, 0.041) | 0.006 | 0.009 |  | 0.024 | (0.008, 0.041) | 0.005 | 0.007 |
|  | HW09 | 0.016 | (-0.002, 0.033) | 0.076 | 0.091 |  | 0.020 | (0.002, 0.037) | 0.030 | 0.036 |  | 0.020 | (0.002, 0.038) | 0.026 | 0.031 |
|  | HW10 | 0.047 | (0.029, 0.065) | <0.001 | <0.001 |  | 0.050 | (0.032, 0.069) | <0.001 | <0.001 |  | 0.051 | (0.033, 0.069) | <0.001 | <0.001 |
|  | HW11 | 0.056 | (0.037, 0.075) | <0.001 | <0.001 |  | 0.060 | (0.040, 0.080) | <0.001 | <0.001 |  | 0.061 | (0.041, 0.080) | <0.001 | <0.001 |
|  | HW12 | 0.053 | (0.034, 0.072) | <0.001 | <0.001 |  | 0.057 | (0.037, 0.076) | <0.001 | <0.001 |  | 0.057 | (0.037, 0.076) | <0.001 | <0.001 |

This table presents the associations between changes in heatwave (ΔHW) exposure and changes in biological age acceleration (ΔBAA, continuous outcome), evaluated using three generalized linear models.

Model 1: Unadjusted model.

Model 2: Adjusted for baseline covariates, including age, gender, body mass index (BMI), education, economic status, residence, geographic region, marital status, smoking, drinking, depressive symptoms, hypertension, and diabetes.

Model 3: Additionally adjusted for time-varying changes in these covariates.

Heatwave exposure was defined by both the number of heatwave events and cumulative HW days, using 12 threshold-duration definitions. Coefficients (β) represent the estimated change in ΔBAA (years) per unit increase in ΔHW. Results are presented with 95% confidence intervals (CIs), along with raw and false discovery rate (FDR)–adjusted p values.

**Table S10.** Longitudinal association between changes in heatwave exposure and accelerated biological aging across three models.

| HW code | | Model 1 (unadjusted) | | | |  | Model 2 (baseline covariates adjusted) | | | |  | Model 3 (baseline + time-varying covariates adjusted) | | | |
| --- | --- | --- | --- | --- | --- | --- | --- | --- | --- | --- | --- | --- | --- | --- | --- |
|  |  | OR | 95%CI | *p* value | FDR-adjusted  *p* value |  | OR | 95%CI | *p* value | FDR-adjusted  *p* value |  | OR | 95%CI | *p* value | FDR-adjusted  *p* value |
| HW events | |  |  |  |  |  |  |  |  |  |  |  |  |  |  |
|  | HW01 | 1.004 | (0.978, 1.030) | 0.785 | 0.785 |  | 1.009 | (0.983, 1.037) | 0.494 | 0.494 |  | 1.012 | (0.985, 1.040) | 0.381 | 0.381 |
|  | HW02 | 1.017 | (0.981, 1.055) | 0.350 | 0.420 |  | 1.015 | (0.978, 1.053) | 0.444 | 0.484 |  | 1.017 | (0.980, 1.056) | 0.365 | 0.381 |
|  | HW03 | 1.070 | (1.015, 1.128) | 0.012 | 0.018 |  | 1.066 | (1.010, 1.126) | 0.021 | 0.028 |  | 1.066 | (1.009, 1.126) | 0.023 | 0.028 |
|  | HW04 | 1.070 | (1.027, 1.115) | 0.001 | 0.002 |  | 1.087 | (1.041, 1.135) | <0.001 | <0.001 |  | 1.089 | (1.042, 1.137) | <0.001 | <0.001 |
|  | HW05 | 1.175 | (1.117, 1.236) | <0.001 | <0.001 |  | 1.190 | (1.128, 1.255) | <0.001 | <0.001 |  | 1.195 | (1.133, 1.260) | <0.001 | <0.001 |
|  | HW06 | 1.115 | (1.035, 1.200) | 0.004 | 0.007 |  | 1.179 | (1.090, 1.276) | <0.001 | <0.001 |  | 1.185 | (1.095, 1.284) | <0.001 | <0.001 |
|  | HW07 | 1.072 | (1.025, 1.122) | 0.003 | 0.006 |  | 1.083 | (1.030, 1.139) | 0.002 | 0.003 |  | 1.084 | (1.031, 1.141) | 0.002 | 0.003 |
|  | HW08 | 1.070 | (1.013, 1.131) | 0.016 | 0.021 |  | 1.074 | (1.013, 1.138) | 0.016 | 0.024 |  | 1.077 | (1.016, 1.142) | 0.013 | 0.017 |
|  | HW09 | 1.029 | (0.963, 1.099) | 0.395 | 0.431 |  | 1.036 | (0.966, 1.111) | 0.326 | 0.391 |  | 1.084 | (1.031, 1.141) | 0.002 | 0.003 |
|  | HW10 | 1.105 | (1.047, 1.166) | <0.001 | <0.001 |  | 1.121 | (1.057, 1.188) | <0.001 | <0.001 |  | 1.125 | (1.061, 1.193) | <0.001 | <0.001 |
|  | HW11 | 1.186 | (1.102, 1.276) | <0.001 | <0.001 |  | 1.228 | (1.133, 1.331) | <0.001 | <0.001 |  | 1.238 | (1.142, 1.343) | <0.001 | <0.001 |
|  | HW12 | 1.194 | (1.091, 1.308) | <0.001 | <0.001 |  | 1.219 | (1.107, 1.341) | <0.001 | <0.001 |  | 1.223 | (1.111, 1.347) | <0.001 | <0.001 |
| HW days | |  |  |  |  |  |  |  |  |  |  |  |  |  |  |
|  | HW01 | 1.006 | (0.999, 1.013) | 0.102 | 0.122 |  | 1.009 | (1.001, 1.017) | 0.030 | 0.036 |  | 1.009 | (1.001, 1.017) | 0.023 | 0.028 |
|  | HW02 | 1.008 | (1.000, 1.016) | 0.043 | 0.057 |  | 1.010 | (1.002, 1.019) | 0.021 | 0.028 |  | 1.010 | (1.002, 1.019) | 0.018 | 0.024 |
|  | HW03 | 1.009 | (1.002, 1.017) | 0.014 | 0.024 |  | 1.011 | (1.003, 1.019) | 0.007 | 0.012 |  | 1.011 | (1.003, 1.019) | 0.008 | 0.014 |
|  | HW04 | 1.011 | (1.003, 1.020) | 0.007 | 0.017 |  | 1.016 | (1.007, 1.026) | 0.001 | 0.002 |  | 1.017 | (1.007, 1.026) | 0.001 | 0.002 |
|  | HW05 | 1.015 | (1.007, 1.024) | <0.001 | <0.001 |  | 1.020 | (1.010, 1.029) | <0.001 | <0.001 |  | 1.020 | (1.011, 1.030) | <0.001 | <0.001 |
|  | HW06 | 1.005 | (0.997, 1.014) | 0.199 | 0.199 |  | 1.009 | (1.000, 1.018) | 0.043 | 0.047 |  | 1.010 | (1.001, 1.019) | 0.037 | 0.040 |
|  | HW07 | 1.010 | (1.002, 1.018) | 0.011 | 0.022 |  | 1.013 | (1.004, 1.021) | 0.004 | 0.008 |  | 1.013 | (1.004, 1.022) | 0.003 | 0.006 |
|  | HW08 | 1.009 | (1.001, 1.017) | 0.027 | 0.040 |  | 1.011 | (1.002, 1.019) | 0.011 | 0.016 |  | 1.011 | (1.003, 1.020) | 0.010 | 0.015 |
|  | HW09 | 1.006 | (0.998, 1.015) | 0.127 | 0.139 |  | 1.009 | (1.000, 1.017) | 0.055 | 0.055 |  | 1.009 | (1.000, 1.018) | 0.048 | 0.048 |
|  | HW10 | 1.021 | (1.012, 1.030) | <0.001 | <0.001 |  | 1.024 | (1.014, 1.034) | <0.001 | <0.001 |  | 1.024 | (1.015, 1.034) | <0.001 | <0.001 |
|  | HW11 | 1.024 | (1.014, 1.035) | <0.001 | <0.001 |  | 1.028 | (1.018, 1.039) | <0.001 | <0.001 |  | 1.029 | (1.018, 1.040) | <0.001 | <0.001 |
|  | HW12 | 1.021 | (1.012, 1.032) | <0.001 | <0.001 |  | 1.024 | (1.014, 1.035) | <0.001 | <0.001 |  | 1.025 | (1.014, 1.035) | <0.001 | <0.001 |

This table presents the associations between changes in heatwave (ΔHW) exposure and the risk of accelerated biological aging (ΔBAA ≥ 0), evaluated using three generalized linear models.

Model 1: Unadjusted model.

Model 2: Adjusted for baseline covariates, including age, gender, body mass index (BMI), education, economic status, residence, geographic region, marital status, smoking, drinking, depressive symptoms, hypertension, and diabetes.

Model 3: Additionally adjusted for time-varying changes in these covariates.

Heatwave exposure was defined by both the number of heatwave events and cumulative HW days, using 12 threshold-duration definitions. Odds ratios (ORs) represent the estimated likelihood of accelerated biological aging (ΔBAA ≥ 0) per unit increase in ΔHW. Results are presented with 95% confidence intervals (CIs), along with raw and false discovery rate (FDR)–adjusted p values.

**Table S11.** Associations between changes in heatwave exposure and biological age acceleration in a linear mixed-effects framework.

| ETEs | | Events | | | |  | Days | | | |
| --- | --- | --- | --- | --- | --- | --- | --- | --- | --- | --- |
|  |  | Beta | 95%CI | *p* value | FDR-adjusted *p* value |  | Beta | 95%CI | *p* value | FDR-adjusted *p* value |
| ﻿Heat wave | |  |  |  |  |  |  |  |  |  |
|  | HW01 | -0.005 | (-0.061, 0.050) | 0.850 | 0.850 |  | 0.016 | (0.000, 0.033) | 0.045 | 0.049 |
|  | HW02 | 0.019 | (-0.057, 0.095) | 0.625 | 0.682 |  | 0.023 | (0.005, 0.041) | 0.011 | 0.014 |
|  | HW03 | 0.111 | (0.001, 0.221) | 0.048 | 0.064 |  | 0.026 | (0.009, 0.042) | 0.003 | 0.005 |
|  | HW04 | 0.185 | (0.097, 0.273) | <0.001 | <0.001 |  | 0.034 | (0.015, 0.053) | <0.001 | 0.001 |
|  | HW05 | 0.363 | (0.261, 0.465) | <0.001 | <0.001 |  | 0.041 | (0.022, 0.060) | <0.001 | <0.001 |
|  | HW06 | 0.297 | (0.139, 0.454) | <0.001 | 0.001 |  | 0.017 | (-0.002, 0.035) | 0.074 | 0.074 |
|  | HW07 | 0.146 | (0.043, 0.249) | 0.005 | 0.009 |  | 0.026 | (0.009, 0.043) | 0.003 | 0.005 |
|  | HW08 | 0.135 | (0.016, 0.254) | 0.026 | 0.038 |  | 0.023 | (0.006, 0.040) | 0.007 | 0.011 |
|  | HW09 | 0.053 | (-0.092, 0.197) | 0.477 | 0.572 |  | 0.019 | (0.001, 0.036) | 0.040 | 0.048 |
|  | HW10 | 0.215 | (0.098, 0.332) | <0.001 | 0.001 |  | 0.05 | (0.032, 0.068) | <0.001 | <0.001 |
|  | HW11 | 0.425 | (0.263, 0.587) | <0.001 | <0.001 |  | 0.059 | (0.040, 0.079) | <0.001 | <0.001 |
|  | HW12 | 0.521 | (0.330, 0.712) | <0.001 | <0.001 |  | 0.055 | (0.036, 0.075) | <0.001 | <0.001 |

This table summarizes the associations between changes in heatwave (ΔHW) exposure and changes in biological age acceleration (ΔBAA), estimated using linear mixed-effects models with area-level random intercepts (northern/southern × rural/urban) to account for geographic clustering. Both the number of heatwave events and cumulative heatwave days were evaluated across 12 HW definitions. All models were adjusted for baseline covariates and their time-varying changes. Results are presented as beta coefficients with 95% confidence intervals (CIs). Raw and false discovery rate (FDR)–adjusted p values are reported to address multiple testing.

**Table S12.** Association between changes in heatwave exposure and biological age acceleration after excluding potential outliers (|ΔBAA| > 10 year).

| HW code | Events | | | |  | Days | | | |
| --- | --- | --- | --- | --- | --- | --- | --- | --- | --- |
|  | Beta | 95%CI | *p* value | FDR-adjusted *p* value |  | Beta | 95%CI | *p* value | FDR-adjusted *p* value |
| HW01 | 0.025 | (-0.025, 0.075) | 0.323 | 0.659 |  | 0.024 | (0.010, 0.038) | 0.001 | 0.136 |
| HW02 | 0.039 | (-0.030, 0.107) | 0.267 | 0.588 |  | 0.028 | (0.012, 0.044) | <0.001 | 0.108 |
| HW03 | 0.131 | (0.033, 0.229) | 0.009 | 0.093 |  | 0.029 | (0.014, 0.043) | <0.001 | 0.068 |
| HW04 | 0.181 | (0.103, 0.259) | <0.001 | <0.001 |  | 0.042 | (0.025, 0.058) | <0.001 | 0.009 |
| HW05 | 0.345 | (0.254, 0.436) | <0.001 | <0.001 |  | 0.047 | (0.031, 0.064) | <0.001 | 0.009 |
| HW06 | 0.339 | (0.198, 0.479) | <0.001 | <0.001 |  | 0.027 | (0.010, 0.043) | 0.001 | 0.104 |
| HW07 | 0.205 | (0.113, 0.296) | <0.001 | 0.018 |  | 0.033 | (0.018, 0.048) | <0.001 | 0.104 |
| HW08 | 0.201 | (0.096, 0.307) | <0.001 | 0.112 |  | 0.029 | (0.014, 0.044) | <0.001 | 0.160 |
| HW09 | 0.157 | (0.028, 0.287) | 0.017 | 0.502 |  | 0.026 | (0.010, 0.041) | 0.001 | 0.313 |
| HW10 | 0.230 | (0.126, 0.335) | <0.001 | 0.014 |  | 0.050 | (0.034, 0.066) | <0.001 | 0.009 |
| HW11 | 0.407 | (0.262, 0.551) | <0.001 | <0.001 |  | 0.058 | (0.041, 0.075) | <0.001 | 0.009 |
| HW12 | 0.430 | (0.260, 0.600) | <0.001 | 0.183 |  | 0.052 | (0.035, 0.069) | <0.001 | 0.053 |

This table presents the associations between changes in heatwave exposure (ΔHW) and changes in biological age acceleration (ΔBAA), after excluding individuals with extreme ΔBAA values (|ΔBAA| > 10 years). A total of 2,255 participants were included in the analysis. Generalized linear models were adjusted for baseline covariates and time-varying changes, consistent with the primary analysis. Beta coefficients represent the estimated change in ΔBAA (years) per unit increase in heatwave events or days. Results include 95% confidence intervals (CIs), raw p values, and false discovery rate (FDR)–adjusted p values.

**Table S13.** Association between changes in heatwave exposure and biological age acceleration after excluding potential outliers (|ΔBAA| > 5 year).

| HW code | Events | | | |  | Days | | | |
| --- | --- | --- | --- | --- | --- | --- | --- | --- | --- |
|  | Beta | 95%CI | *p* value | FDR-adjusted *p* value |  | Beta | 95%CI | *p* value | FDR-adjusted *p* value |
| HW01 | 0.009 | (-0.030, 0.047) | 0.659 | 0.659 |  | 0.009 | (-0.002, 0.020) | 0.113 | 0.136 |
| HW02 | 0.017 | (-0.036, 0.069) | 0.539 | 0.588 |  | 0.011 | (-0.001, 0.022) | 0.081 | 0.108 |
| HW03 | 0.074 | (-0.001, 0.149) | 0.054 | 0.093 |  | 0.012 | (0.001, 0.023) | 0.034 | 0.068 |
| HW04 | 0.109 | (0.050, 0.169) | <0.001 | <0.001 |  | 0.019 | (0.006, 0.032) | 0.003 | 0.009 |
| HW05 | 0.2 | (0.130, 0.270) | <0.001 | <0.001 |  | 0.022 | (0.010, 0.035) | 0.001 | 0.009 |
| HW06 | 0.223 | (0.116, 0.330) | <0.001 | <0.001 |  | 0.012 | (-0.001, 0.024) | 0.066 | 0.104 |
| HW07 | 0.093 | (0.023, 0.164) | 0.009 | 0.018 |  | 0.011 | (-0.001, 0.022) | 0.069 | 0.104 |
| HW08 | 0.073 | (-0.007, 0.154) | 0.075 | 0.112 |  | 0.009 | (-0.003, 0.020) | 0.147 | 0.16 |
| HW09 | 0.041 | (-0.058, 0.14) | 0.418 | 0.502 |  | 0.006 | (-0.006, 0.018) | 0.313 | 0.313 |
| HW10 | 0.113 | (0.033, 0.193) | 0.006 | 0.014 |  | 0.019 | (0.006, 0.031) | 0.003 | 0.009 |
| HW11 | 0.198 | (0.088, 0.309) | <0.001 | <0.001 |  | 0.022 | (0.008, 0.035) | 0.002 | 0.009 |
| HW12 | 0.1 | (-0.032, 0.232) | 0.137 | 0.183 |  | 0.016 | (0.002, 0.029) | 0.022 | 0.053 |

This table presents the associations between changes in heatwave exposure (ΔHW) and changes in biological age acceleration (ΔBAA), after excluding individuals with extreme ΔBAA values (|ΔBAA| > 5 years). A total of 1,798 participants were included in the analysis. Generalized linear models were adjusted for baseline covariates and time-varying changes, consistent with the primary analysis. Beta coefficients represent the estimated change in ΔBAA (years) per unit increase in heatwave events or days. Results include 95% confidence intervals (CIs), raw p values, and false discovery rate (FDR)–adjusted p values.

**Table S14.** Association between changes in heatwave exposure and accelerated biological aging after excluding minimal change observations (|ΔBAA| < 1 year).

| HW code | Events | | | |  | Days | | | |
| --- | --- | --- | --- | --- | --- | --- | --- | --- | --- |
|  | OR | 95%CI | *p* value | FDR-adjusted *p* value |  | OR | 95%CI | *p* value | FDR-adjusted *p* value |
| HW01 | 1.004 | (0.973, 1.035) | 0.814 | 0.814 |  | 1.009 | (1.000, 1.018) | 0.063 | 0.063 |
| HW02 | 1.010 | (0.968, 1.054) | 0.633 | 0.691 |  | 1.011 | (1.001, 1.021) | 0.036 | 0.043 |
| HW03 | 1.062 | (0.998, 1.131) | 0.058 | 0.077 |  | 1.012 | (1.003, 1.022) | 0.011 | 0.019 |
| HW04 | 1.098 | (1.045, 1.154) | <0.001 | <0.001 |  | 1.020 | (1.009, 1.032) | <0.001 | <0.001 |
| HW05 | 1.231 | (1.157, 1.310) | <0.001 | <0.001 |  | 1.025 | (1.014, 1.037) | <0.001 | <0.001 |
| HW06 | 1.218 | (1.112, 1.335) | <0.001 | <0.001 |  | 1.012 | (1.002, 1.022) | 0.025 | 0.033 |
| HW07 | 1.097 | (1.035, 1.163) | 0.002 | 0.003 |  | 1.015 | (1.005, 1.025) | 0.004 | 0.008 |
| HW08 | 1.078 | (1.008, 1.153) | 0.029 | 0.044 |  | 1.012 | (1.003, 1.022) | 0.013 | 0.020 |
| HW09 | 1.034 | (0.954, 1.121) | 0.412 | 0.494 |  | 1.010 | (1.000, 1.020) | 0.057 | 0.062 |
| HW10 | 1.129 | (1.056, 1.207) | <0.001 | <0.001 |  | 1.029 | (1.017, 1.040) | <0.001 | <0.001 |
| HW11 | 1.247 | (1.137, 1.368) | <0.001 | <0.001 |  | 1.034 | (1.021, 1.047) | <0.001 | <0.001 |
| HW12 | 1.244 | (1.114, 1.389) | <0.001 | <0.001 |  | 1.029 | (1.017, 1.042) | <0.001 | <0.001 |

This table presents the associations between changes in heatwave (ΔHW) exposure and the likelihood of accelerated biological aging, defined as ΔBAA ≥ 1 year. Observations with minimal changes in biological age (|ΔBAA| < 1) were excluded to focus on clinically meaningful shifts. Odds ratios (ORs) with 95% confidence intervals (CIs), p values, and false discovery rate (FDR)–adjusted p values are shown for both HW event counts and cumulative HW days. All models were adjusted for baseline covariates and time-varying changes, consistent with the primary analysis.

**Table S15**. Statistical significance in associations between changes in heatwave exposure and changes in biological age–related biomarkers.

| HW code | | PLT | |  | CRP | |  | HbA1c | |  | SCR | |  | BUN | |  | TC | |  | TG | |  | SBP | |
| --- | --- | --- | --- | --- | --- | --- | --- | --- | --- | --- | --- | --- | --- | --- | --- | --- | --- | --- | --- | --- | --- | --- | --- | --- |
|  |  | Raw *p* | FDR *p* |  | Raw *p* | FDR *p* |  | Raw *p* | FDR *p* |  | Raw *p* | FDR *p* |  | Raw *p* | FDR *p* |  | Raw *p* | FDR *p* |  | Raw *p* | FDR *p* |  | Raw *p* | FDR *p* |
| HW Events | |  |  |  |  |  |  |  |  |  |  |  |  |  |  |  |  |  |  |  |  |  |  |  |
|  | HW01 | 0.038 | 0.194 |  | 0.024 | 0.091 |  | 0.445 | 0.445 |  | 0.306 | 0.367 |  | 0.428 | 0.514 |  | 0.002 | 0.002 |  | 0.619 | 0.825 |  | 0.626 | 0.751 |
|  | HW02 | 0.343 | 0.515 |  | 0.045 | 0.108 |  | 0.120 | 0.131 |  | 0.136 | 0.204 |  | 0.575 | 0.575 |  | <0.001 | <0.001 |  | 0.057 | 0.341 |  | 0.361 | 0.559 |
|  | HW03 | 0.397 | 0.529 |  | 0.157 | 0.236 |  | <0.001 | <0.001 |  | 0.627 | 0.627 |  | 0.024 | 0.047 |  | <0.001 | <0.001 |  | 0.833 | 0.909 |  | 0.815 | 0.815 |
|  | HW04 | 0.694 | 0.757 |  | <0.001 | 0.001 |  | 0.044 | 0.052 |  | 0.032 | 0.064 |  | 0.028 | 0.048 |  | <0.001 | <0.001 |  | 0.089 | 0.357 |  | 0.373 | 0.559 |
|  | HW05 | 0.064 | 0.194 |  | <0.001 | 0.002 |  | 0.001 | 0.002 |  | <0.001 | 0.001 |  | <0.001 | 0.001 |  | <0.001 | <0.001 |  | 0.277 | 0.475 |  | 0.290 | 0.559 |
|  | HW06 | 0.503 | 0.604 |  | 0.030 | 0.091 |  | <0.001 | 0.001 |  | 0.046 | 0.079 |  | 0.267 | 0.356 |  | <0.001 | <0.001 |  | 0.155 | 0.465 |  | 0.435 | 0.580 |
|  | HW07 | 0.060 | 0.194 |  | 0.098 | 0.196 |  | 0.020 | 0.027 |  | 0.014 | 0.032 |  | 0.001 | 0.007 |  | 0.001 | 0.001 |  | 0.009 | 0.112 |  | 0.004 | 0.043 |
|  | HW08 | 0.124 | 0.258 |  | 0.499 | 0.562 |  | <0.001 | <0.001 |  | <0.001 | <0.001 |  | 0.005 | 0.015 |  | 0.001 | 0.001 |  | 0.207 | 0.475 |  | 0.047 | 0.189 |
|  | HW09 | 0.129 | 0.258 |  | 0.695 | 0.695 |  | <0.001 | <0.001 |  | <0.001 | <0.001 |  | 0.521 | 0.568 |  | <0.001 | <0.001 |  | 0.381 | 0.572 |  | 0.020 | 0.123 |
|  | HW10 | 0.053 | 0.194 |  | 0.515 | 0.562 |  | <0.001 | <0.001 |  | 0.626 | 0.627 |  | 0.002 | 0.007 |  | <0.001 | <0.001 |  | 0.273 | 0.475 |  | 0.161 | 0.483 |
|  | HW11 | 0.163 | 0.279 |  | 0.115 | 0.197 |  | <0.001 | <0.001 |  | 0.185 | 0.247 |  | 0.006 | 0.015 |  | <0.001 | <0.001 |  | 0.999 | 0.999 |  | 0.749 | 0.815 |
|  | HW12 | 0.806 | 0.806 |  | 0.401 | 0.535 |  | <0.001 | <0.001 |  | <0.001 | <0.001 |  | 0.089 | 0.133 |  | 0.062 | 0.062 |  | 0.729 | 0.875 |  | 0.230 | 0.552 |
| HW Days | |  |  |  |  |  |  |  |  |  |  |  |  |  |  |  |  |  |  |  |  |  |  |  |
|  | HW01 | 0.001 | 0.009 |  | 0.162 | 0.877 |  | <0.001 | <0.001 |  | 0.011 | 0.023 |  | 0.816 | 0.979 |  | <0.001 | <0.001 |  | 0.640 | 0.781 |  | 0.001 | 0.003 |
|  | HW02 | 0.004 | 0.023 |  | 0.290 | 0.877 |  | <0.001 | <0.001 |  | 0.126 | 0.168 |  | 0.981 | 0.981 |  | <0.001 | <0.001 |  | 0.988 | 0.988 |  | <0.001 | 0.001 |
|  | HW03 | 0.070 | 0.094 |  | 0.703 | 0.877 |  | <0.001 | <0.001 |  | 0.009 | 0.020 |  | 0.182 | 0.388 |  | <0.001 | <0.001 |  | 0.200 | 0.781 |  | 0.002 | 0.007 |
|  | HW04 | 0.034 | 0.090 |  | 0.218 | 0.877 |  | <0.001 | <0.001 |  | 0.050 | 0.086 |  | 0.360 | 0.540 |  | <0.001 | <0.001 |  | 0.716 | 0.781 |  | 0.017 | 0.052 |
|  | HW05 | 0.009 | 0.035 |  | 0.379 | 0.877 |  | <0.001 | <0.001 |  | 0.103 | 0.154 |  | 0.169 | 0.388 |  | <0.001 | <0.001 |  | 0.487 | 0.781 |  | 0.094 | 0.161 |
|  | HW06 | 0.063 | 0.094 |  | 0.821 | 0.877 |  | <0.001 | <0.001 |  | <0.001 | <0.001 |  | 0.780 | 0.979 |  | <0.001 | <0.001 |  | 0.448 | 0.781 |  | 0.055 | 0.110 |
|  | HW07 | 0.037 | 0.090 |  | 0.834 | 0.877 |  | <0.001 | <0.001 |  | 0.823 | 0.898 |  | 0.194 | 0.388 |  | <0.001 | 0.001 |  | 0.199 | 0.781 |  | 0.054 | 0.110 |
|  | HW08 | 0.059 | 0.094 |  | 0.877 | 0.877 |  | <0.001 | <0.001 |  | 0.919 | 0.919 |  | 0.311 | 0.533 |  | 0.001 | 0.001 |  | 0.442 | 0.781 |  | 0.130 | 0.173 |
|  | HW09 | 0.066 | 0.094 |  | 0.753 | 0.877 |  | <0.001 | <0.001 |  | 0.509 | 0.611 |  | 0.951 | 0.981 |  | <0.001 | 0.001 |  | 0.573 | 0.781 |  | 0.121 | 0.173 |
|  | HW10 | 0.272 | 0.326 |  | 0.706 | 0.877 |  | <0.001 | <0.001 |  | <0.001 | <0.001 |  | 0.011 | 0.103 |  | 0.002 | 0.002 |  | 0.408 | 0.781 |  | 0.701 | 0.701 |
|  | HW11 | 0.377 | 0.411 |  | 0.563 | 0.877 |  | <0.001 | <0.001 |  | <0.001 | <0.001 |  | 0.017 | 0.103 |  | 0.002 | 0.003 |  | 0.598 | 0.781 |  | 0.416 | 0.499 |
|  | HW12 | 0.774 | 0.774 |  | 0.798 | 0.877 |  | <0.001 | <0.001 |  | <0.001 | <0.001 |  | 0.060 | 0.238 |  | 0.040 | 0.040 |  | 0.679 | 0.781 |  | 0.583 | 0.636 |

This table presents the associations between changes in heatwave exposure (ΔHW) and changes in key biomarkers related to biological aging, including platelet count (PLT), C-reactive protein (CRP), glycated hemoglobin (HbA1c), serum creatinine (SCR), blood urea nitrogen (BUN), total cholesterol (TC), triglycerides (TG), and systolic blood pressure (SBP). Both the number of heatwave events and cumulative heatwave days were assessed across 12 definitions. Results are reported as raw and false discovery rate (FDR)–adjusted p values. Generalized linear models were adjusted for baseline covariates and time-varying changes, consistent with the main analysis. Associations with FDR-adjusted p < 0.05 were considered statistically significant.

**Table S16**. Association between changes in heatwave exposure and changes in HbA1c and total cholesterol.

| HW code | | HbA1c | | |  |  | Total Cholesterol | | |  |
| --- | --- | --- | --- | --- | --- | --- | --- | --- | --- | --- |
|  |  | Beta | 95%CI | *p* value | FDR-adjusted *p* value |  | Beta | 95%CI | *p* value | FDR-adjusted *p* value |
| HW Events | |  |  |  |  |  |  |  |  |  |
|  | HW01 | -0.004 | (-0.013, 0.006) | 0.445 | 0.445 |  | 0.650 | (0.236, 1.065) | 0.002 | 0.002 |
|  | HW02 | 0.010 | (-0.003, 0.022) | 0.120 | 0.131 |  | 1.281 | (0.710, 1.851) | <0.001 | <0.001 |
|  | HW03 | 0.034 | (0.016, 0.052) | <0.001 | <0.001 |  | 1.982 | (1.170, 2.794) | <0.001 | <0.001 |
|  | HW04 | 0.015 | (0, 0.029) | 0.044 | 0.052 |  | 1.793 | (1.151, 2.435) | <0.001 | <0.001 |
|  | HW05 | 0.028 | (0.011, 0.045) | 0.001 | 0.002 |  | 2.394 | (1.626, 3.162) | <0.001 | <0.001 |
|  | HW06 | 0.046 | (0.020, 0.072) | <0.001 | 0.001 |  | 3.292 | (2.128, 4.456) | <0.001 | <0.001 |
|  | HW07 | 0.019 | (0.003, 0.036) | 0.020 | 0.026 |  | 1.238 | (0.494, 1.982) | 0.001 | 0.001 |
|  | HW08 | 0.038 | (0.019, 0.058) | <0.001 | <0.001 |  | 1.547 | (0.666, 2.428) | 0.001 | 0.001 |
|  | HW09 | 0.060 | (0.037, 0.083) | <0.001 | <0.001 |  | 2.369 | (1.314, 3.424) | <0.001 | <0.001 |
|  | HW10 | 0.061 | (0.042, 0.080) | <0.001 | <0.001 |  | 1.745 | (0.877, 2.614) | <0.001 | <0.001 |
|  | HW11 | 0.090 | (0.064, 0.116) | <0.001 | <0.001 |  | 2.558 | (1.364, 3.752) | <0.001 | <0.001 |
|  | HW12 | 0.085 | (0.054, 0.116) | <0.001 | <0.001 |  | 1.350 | (-0.064, 2.765) | 0.061 | 0.061 |
| HW Days | |  |  |  |  |  |  |  |  |  |
|  | HW01 | 0.006 | (0.003, 0.008) | <0.001 | <0.001 |  | 0.242 | (0.129, 0.355) | <0.001 | <0.001 |
|  | HW02 | 0.009 | (0.006, 0.011) | <0.001 | <0.001 |  | 0.301 | (0.176, 0.426) | <0.001 | <0.001 |
|  | HW03 | 0.009 | (0.006, 0.011) | <0.001 | <0.001 |  | 0.238 | (0.118, 0.358) | <0.001 | <0.001 |
|  | HW04 | 0.009 | (0.006, 0.012) | <0.001 | <0.001 |  | 0.356 | (0.225, 0.487) | <0.001 | <0.001 |
|  | HW05 | 0.009 | (0.006, 0.012) | <0.001 | <0.001 |  | 0.354 | (0.221, 0.487) | <0.001 | <0.001 |
|  | HW06 | 0.008 | (0.005, 0.011) | <0.001 | <0.001 |  | 0.259 | (0.128, 0.390) | <0.001 | <0.001 |
|  | HW07 | 0.006 | (0.004, 0.009) | <0.001 | <0.001 |  | 0.224 | (0.099, 0.349) | <0.001 | 0.001 |
|  | HW08 | 0.007 | (0.004, 0.009) | <0.001 | <0.001 |  | 0.216 | (0.091, 0.341) | 0.001 | 0.001 |
|  | HW09 | 0.007 | (0.005, 0.010) | <0.001 | <0.001 |  | 0.240 | (0.110, 0.369) | <0.001 | 0.001 |
|  | HW10 | 0.009 | (0.006, 0.012) | <0.001 | <0.001 |  | 0.214 | (0.078, 0.349) | 0.002 | 0.002 |
|  | HW11 | 0.009 | (0.006, 0.013) | <0.001 | <0.001 |  | 0.230 | (0.082, 0.378) | 0.002 | 0.003 |
|  | HW12 | 0.008 | (0.005, 0.011) | <0.001 | <0.001 |  | 0.154 | (0.007, 0.300) | 0.039 | 0.039 |

This table presents the associations between changes in heatwave exposure (ΔHW)—measured as the number of events and cumulative days—and changes in glycated hemoglobin (HbA1c, % of total hemoglobin) and total cholesterol (TC, mg/dL). Analyses were conducted using generalized linear models adjusted for the same baseline and time-varying covariates as in the primary analysis. Results are shown as beta coefficients per unit increase in ΔHW, with 95% confidence intervals (CIs), along with raw and false discovery rate (FDR)–adjusted p values. Statistically significant associations (FDR-adjusted p < 0.05) indicate robust relationships between specific HW definitions and biomarker changes.
